# A bioactivity-leveling approach for component-specific evaluation in natural product extracts: Optimizing SARS-CoV-2 antiviral screening by controlling for pheophorbide A

**DOI:** 10.1371/journal.pone.0355466

**Published:** 2026-08-24

**Authors:** Corinna Urmann, Yvonne Gmach, Niklas Hofer, Nina Vahekeni, Jonas Stehlin, Yannick Geissmann, Andreas Lardos, Olivier Engler, Evelyn Wolfram

**Affiliations:** 1 Organic-analytical Chemistry, Weihenstephan-Triesdorf University of Applied Sciences, Straubing, Germany; 2 TUM Campus Straubing for Biotechnology and Sustainability, Technical University of Munich, Straubing, Germany; 3 Natural Product Chemistry and Phytopharmacy Group, Institute of Chemistry and Biotechnology, School of Life Sciences and Facility Management, ZHAW Zurich University of Applied Sciences, Wädenswil, Switzerland; 4 Spiez Laboratory, Federal Office for Civil Protection, Spiez, Switzerland; University of Buea, CAMEROON

## Abstract

The emergence of SARS-CoV-2 has intensified the search for novel antiviral agents, with plants representing a promising source due to the documented antiviral properties. Pheophorbides, chlorophyll breakdown products, have been demonstrated to impede viral entry of enveloped viruses. However, the frequent occurrence of pheophorbide A in plant extracts presents a challenge for antiviral screening, as it may obscure the discovery of novel antiviral compounds. The objective of this study was therefore leveling out the influence of pheophorbide A in an *in vitro* virus pretreatment assay, to prioritize extracts and fractions and support the identification of additional antiviral compounds in plant extracts. Using different experimental derived examples, the limitations and advantages of the strategy presented are discussed. Assay-derived protective effects were interpreted in relation to pheophorbide A quantification, allowing six out of 15 plant species to be prioritized for further investigations. Qualitative comparison of metabolite profiles of fractions with the extent of protective effect supported the prioritization process and led to the identification of candidate compounds potentially contributing to the observed protective effect in *Primula veris* L. beyond pheophorbide A. Overall, the results highlight the potential value of the proposed strategy for revealing bioactivity beyond known predominant effectors and suggest an applicability in natural product–based screening approaches.

## Introduction

In consequence of the COVID-19 pandemic, which was caused by SARS-CoV-2, it has become apparent that the development of active compounds against hitherto unknown viruses will be a public necessity in the future. Plants can serve as a source of such active compounds, particularly given the existing use of plants and plant-derived natural products in the treatment of respiratory diseases [1,2]. The antiviral activities of plant-derived natural products have been widely researched and are the topic of numerous reviews [1,3,4]. A search of the PubMed database for publications using the query “antiviral + plant extract” yielded a total of 7922 hits, with almost a quarter (1778) of these being published since 2020. A more focused search for plant extracts and SARS-CoV-2 (SARS-CoV-2 + Plant extract) returned a total of 809 results [5].

Pathogenic viruses can be classified into two principal groups: non-enveloped and enveloped viruses. Non-enveloped viruses demonstrate greater resistance to chemical and physical processes and other environmental influences, and the process of exit from the host cell is accomplished by destruction [6,7]. In contrast, viruses that are enveloped are characterized by the presence of a lipid bilayer envelope, which serves to protect the viral genome and allows the virus to leave the host cell without lysis. The envelope is also the reason why inactivating the virus is more susceptible to soaps, detergents, and mild disinfectants [6,7]. Examples of human pathogenic enveloped viruses include the herpes simplex virus [8], influenza viruses [9], the Zaire Ebola virus [10], and also SARS-CoV-2 [11].

A variety of natural compounds and extracts with antiviral activities have been identified, depending on the specific source plant and the solvent and process used for extraction. The latter include plant derived compounds and extracts that inhibit virus entry, prevent or hinder the virus from attachment to the cells, inhibit replication enzymes and block virus release [3,4].

There are a plethora of *in vitro* assays for the screening of antiviral compounds. However, these can be broadly classified into three distinct protocols: [12] 1) Virus pretreatment assay: The virus is incubated with the compounds of interest for a designated time period. Subsequently, the virus suspension is administered to the cells for infection. This approach is also referred to as virucidal activity, as it directly influences the virus particles. 2) Cotreatment assay: cells are treated in parallel with the compound and the virus and 3) Postinfection treatment assay: Cells are infected with virus and the compound is added at a specified point in time in order to study the post-infective antiviral activity.

The use of a pretreatment assay in antiviral research offers the benefits of determining the preventive efficacy and testing for the effects on both the initial and later stages of the viral life cycle [13]. The extracts or compounds identified as protective in a pretreatment assay can demonstrate efficacy against a diverse array of viruses and may also exhibit prolonged effects, rendering these extracts and compounds derived from plants suitable for prophylactic use [14].

In some studies of plant extracts, chlorophyll break-down products have been identified, especially pheophorbides (alternatively spelled phaeophorbides), which have been shown to inhibit viral entry by interacting directly with the virus particle of enveloped viruses [12,15–17]. There are a number of different breakdown products of chlorophyll, resulting in pheophorbide A and B, as well as the ethyl esters [12,18–21]. Furthermore, harderoporphyrin and pyropheophorbides A and B, in addition to red chlorophyll catabolites (open structure), are known [12,18,19].

One advantage of pheophorbide A as an antiviral agent being a breakdown product of chlorophyll, might be that it is a highly available ubiquitous natural product and can be derived from plant material as well as from other sources such as microalgae [22]. Virus deactivators can therefore be plant extracts with increased pheophorbide A content, regardless of the plant species [22]. Conversely, the presence of pheophorbide A presents a significant challenge in downstream screening efforts involving plant-derived natural extracts intended for the discovery of antiviral compounds against enveloped viruses. The frequent occurrence of pheophorbides may lead to repeated rediscovery of these known compounds or to the premature exclusion of extracts and fractions in which pheophorbides are detected by LC-UV/Vis or LC-MS analysis. Dereplication in natural product research is inherently resource-intensive, both in terms of time and cost, due to several major challenges – most notably, the chemical complexity of natural product mixtures, the requirement for sophisticated analytical instrumentation, and the need for highly specialized expertise [23]. Furthermore, the early elimination of extracts based on known constituents carries the risk of overlooking co-occurring, highly potent bioactive compounds.

During the authors’ ongoing screening on antiviral plant extracts and natural products [24], the mentioned challenges due to pheophorbide A content became apparent [25] and have led to the following objective of this study: leveling out the influence of pheophorbide A from the screening approach, in order to support the identification of additional compounds with antiviral activity through an integrated strategy combining metabolite profiling and bioactivity studies. Accordingly, the primary focus of this study is the evaluation of the screening method itself, rather than a comprehensive assessment of the antiviral properties of the extracts or compounds identified.

## Results and discussion

**Fig 1:**
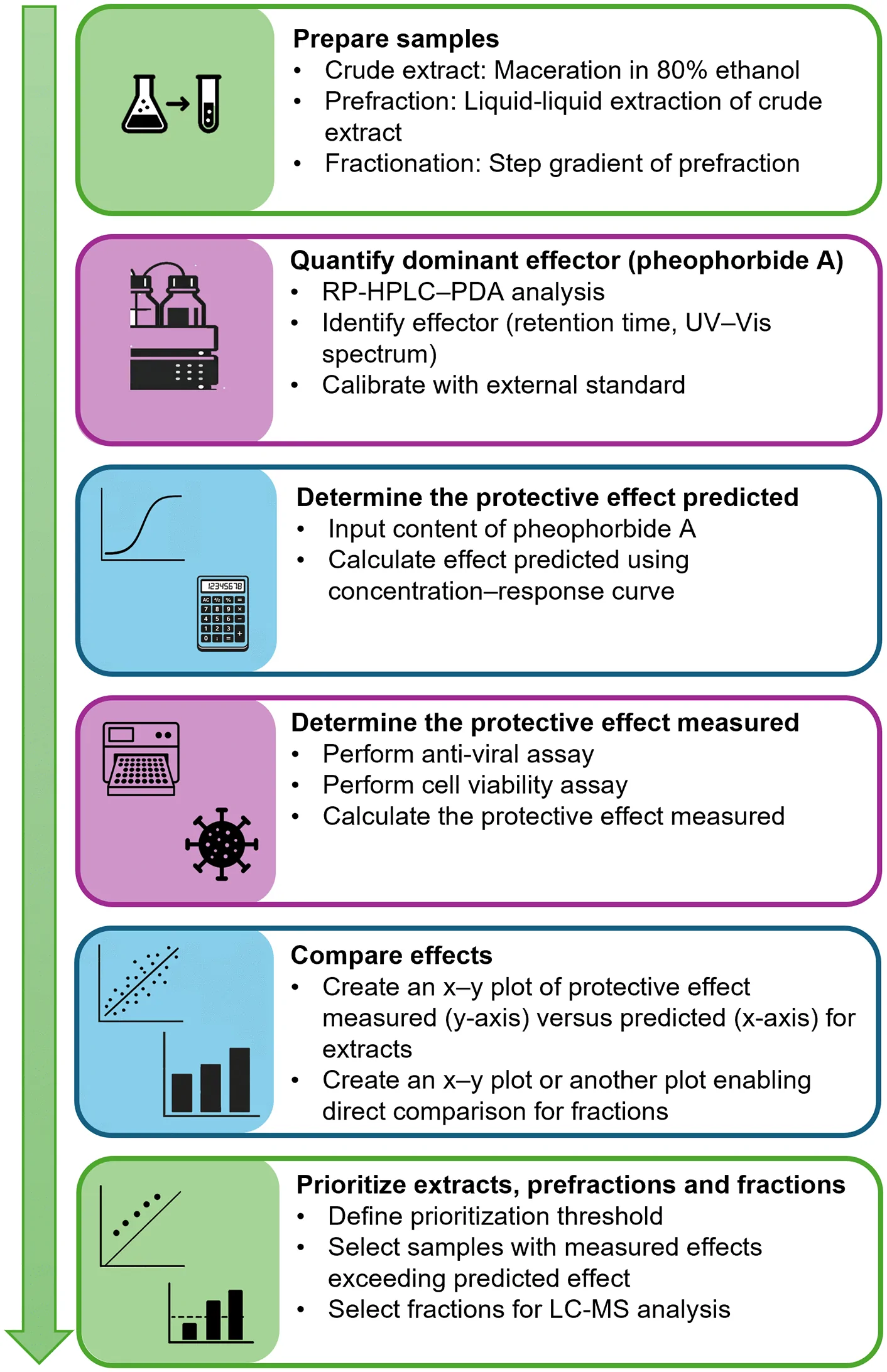
Schematic overview of the bioactivity-leveling strategy, from pheophorbide A quantification and predicted effect calculation to extract prioritization and subsequent fraction analysis.

The proposed strategy is based on prediction of the protective effect of pheophorbide A against SARS-CoV-2, derived from the quantity within extracts and fractions. Subsequently, this predicted effect is associated with the protective effect experimentally measured using the virus pretreatment assay (Fig 1). Comparison of both effects allows an estimation of whether the observed effect can be explained by the presence of pheophorbide A or whether additional bioactive compounds are likely involved. The measured protective effects reflect protection against virus-induced reduction of cell viability in the pretreatment assay and should therefore be interpreted as indirect measures of antiviral activity. All observed relations were interpreted to support prioritization for further investigation and to assess whether the measured effects deviated from the pheophorbide A-based prediction. Following the identification of potential active components, validation was performed using pure compounds and biological replication.

By systematically integrating quantitative chemical profiling with experimentally determined bioactivity data, this approach advances conventional MS-based dereplication strategies. Rather than excluding extracts, prefractions, or fractions solely on the basis of the presence of known antiviral constituents such as pheophorbide A, the method enables identification of samples in which the observed effect exceeds the predicted contribution of the known compound. Such samples can thereby be prioritized for further phytochemical and bioactivity characterization, minimizing the risk of disregarding additional active compounds within complex extract matrices. This is particularly advantageous when experiments have to be performed under demanding conditions, such as in high-containment laboratories requiring full-body protective equipment, where throughput and operational time are limited. In such settings, early prioritization of active extracts using crude material can streamline the workflow before committing to more resource-intensive fractionation steps [26] and higher number of samples to be tested. Accordingly, initiating the screening process with crude extracts can offer significant time and resource savings under such circumstances, even though the advantages of prefractionation are well known [26].

In order to establish a relation between the pheophorbide A content in plant extracts and the antiviral activity in the pretreatment assay, the protective effect of pheophorbide A against SARS-CoV-2 as a function of concentration in the range of 25 ng/mL to 1.5 µg/mL was determined (S1 Fig.). For IC_50_ determination, it is recommended that more than six concentrations are used [27]. However, for the relation established, additional values are required in the range between 0 and 100% inhibition for a more accurate prediction of the protective effect. The effect of twenty concentrations of pheophorbide A were therefore determined in three independent replicates using the aforementioned pretreatment assay in order to ascertain the protective effects of this compound. The IC_50_ value for pheophorbide A was determined to be 25 ng/mL, which is in accordance with the findings of other studies using Vero E6 cells [16]. Subsequently, the pheophorbide A content of extracts and (pre)fractions of the investigated European Medicinal Plants was quantified by LC-PDA (λ = 650 nm) using a calibration curve (c = 0.06 µg/ml to 14.8 µg/mL).

### Prioritization of crude extracts

In a next step, crude extracts derived from different plant species and plant parts (Table 1) were evaluated.

**Table 1:** Investigated plants and plant parts.

Plant code	Plant species	Plant family	Plant part
P1	*Artemisia annua* L.	Asteraceae	aerial part
P2	*Artemisia vulgaris* L.	Asteraceae	aerial part
P3	*Centaurium erythraea* Rafn	Gentianaceae	aerial part
P4	*Cichorium intybus* L.	Asteraceae	aerial part
P5	*Geranium robertianum* L.	Geraniaceae	aerial part
P6	*Melissa officinalis* L.	Lamiaceae	leaves
P7	*Primula veris* L.	Primulaceae	inflorescence including peduncle
P8	*Salix alba* L.	Salicaceae	leaves
P9	*Sambucus nigra* L.	Viburnaceae	leaves
P10	*Stellaria media* (L.) Vill.	Caryophyllaceae	aerial part
P11	*Thymus vulgaris* L.	Lamiaceae	aerial part
P12	*Urtica dioica* L.	Urticaceae	leaves
P13	*Verbascum densiflorum* Bertol.	Scrophulariaceae	flower
P14	*Veronica officinalis* L.	Plantaginaceae	aerial part
P15	*Viola odorata* L.	Violaceae	leaves

The crude extracts of the plants under investigation were tested at five different concentrations in order to determine the antiviral activity. The rationale behind conducting the test at five different concentrations is twofold. First, a concentration that does not affect cell viability is required in order to determine the protective effect afforded (S2, S3, S4 and S5 Tables). Second, the protective effect should show a concentration-dependent trend to support the interpretation of the observed effect. In light of the aforementioned prerequisites, it is of paramount importance to use a range of concentrations and not a single global one for the various plant extracts to allow robust interpretation of observed effect patterns.

**Fig 2:**
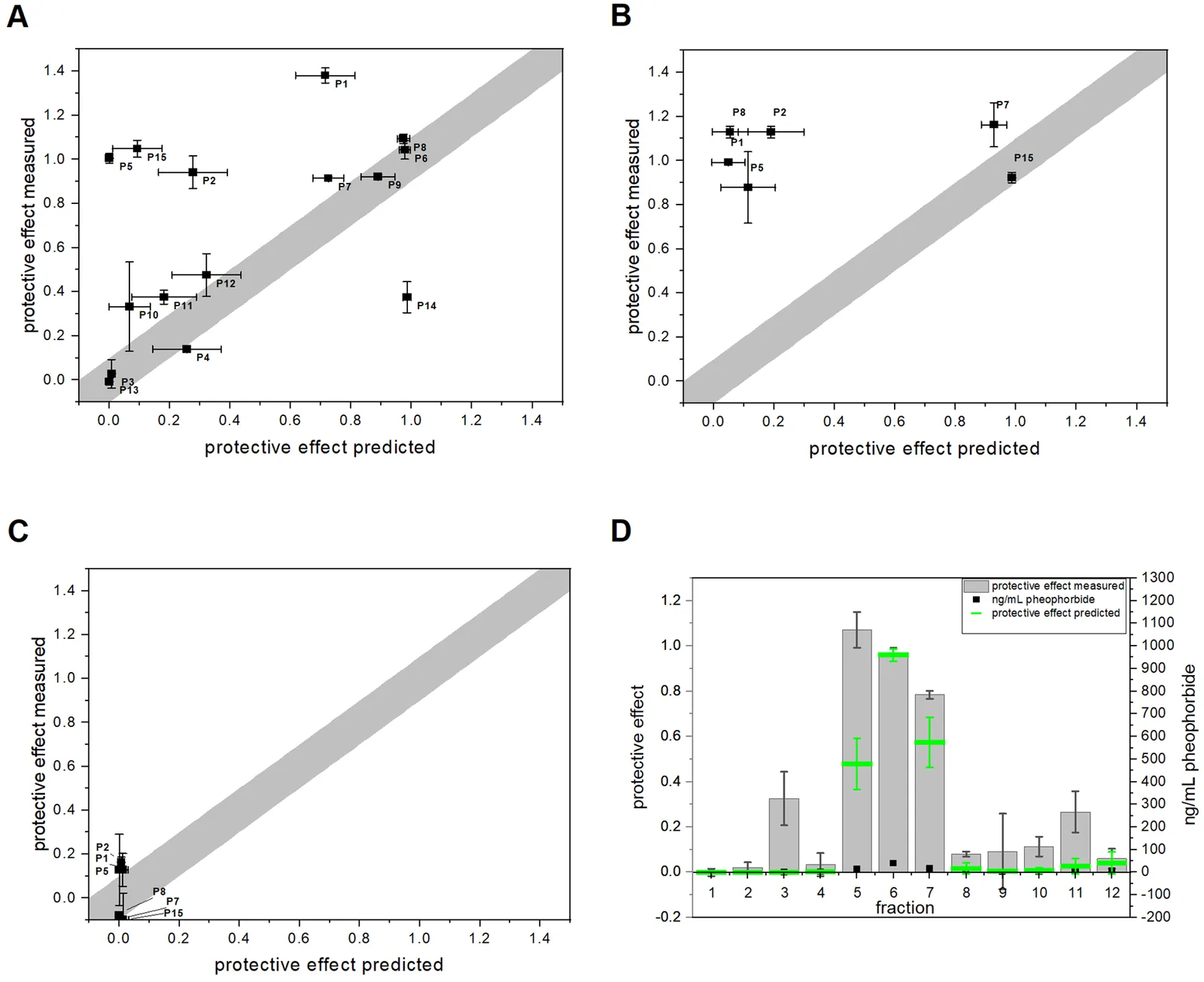
Protective effect predicted (mean±SD, n = 3) versus measured protective effect of A) crude extracts of plant species P1-15; B) organic prefractions of plant species *Artemisia annua* (P1; c = 1.85 µg/mL), *Artemisia vulgaris* (P2; c = 1.85 µg/mL), *Geranium robertianum* (P5; c = 5.56 µg/mL), *Primula veris* (P7; c = 5.56 µg/mL), *Salix alba* (P8; c = 5.56 µg/mL); C) aqueous prefractions of plant species *Artemisia annua* (P1; c = 16.67 µg/mL), *Artemisia vulgaris* (P2; c = 16.67 µg/mL), *Geranium robertianum* (P5; c = 16.67 µg/mL), *Primula veris* (P7; c = 16.67 µg/mL), *Salix alba* (P8; c = 16.67 µg/mL), and *Viola odorata* (P15; c = 16.67 µg/mL), including the screening tolerance range (grey band); D) Protective effect measured (grey bars,) and protective effect predicted (green lines, n = 3, mean ± SD) from pheophorbide A content (black squares) of *Primula veris* (P7) fractions (c = 5.56 µg/mL).

Using the concentration-response curves of pheophorbide A (S1 Fig.) established in three independent experiments, the predicted protective effects based on the determined pheophorbide A content (Table 2) were calculated and compared to the protective effect measured in the pretreatment assay (Fig 2A) using one-way ANOVA followed by Bonferroni post hoc test. The 10% screening tolerance range (grey band, Fig 2A-2C) was introduced to facilitate visualization of prioritization of extracts during the screening workflow. The selected range was based on the observed technical variability of the assay, as reflected by the coefficients of variation (CV) determined for the cell control (6.4%), positive control (4.3%), and solvent control (10.9%). The mean CV across these controls (7.2%) indicates that deviations in the range of ±10% are within the expected assay variability, thereby supporting the chosen tolerance range.

**Table 2:** Concentration of crude extracts applied in the pretreatment assay, corresponding cell viability, pheophorbide A content and comparison of measured and predicted effect.

Plant code	c (µg/mL)	Cell viability (% of untreated control)	Pheophorbide A (µg/mL)	Measured effect (mean±SD)*	Predicted effect (mean±SD, n = 3)	p value	Result of comparison measured and predicted protective effect
P1	5.56	122%	0.038	1.380 ± 0.031	0.715 ± 0.098	0.003	Significant difference
P2	5.56	110%	0.021	0.941 ± 0.074	0.277 ± 0.114	0.006	Significant difference
P3	16.67	94%	n.d.	0.028 ± 0.065	n.c.	0.606	No significant difference
P4	100.00	69%	0.021	0.139 ± 0.004	0.257 ± 0.113	0.583	No significant difference
P5	5.56	99%	n.d.	1.005 ± 0.022	n.c.	<0.001	Significant difference
P6	50.00	100%	0.099	1.043 ± 0.042	0.978 ± 0.018	0.087	No significant difference
P7	50.00	100%	0.037	0.915 ± 0.008	0.724 ± 0.051	0.016	Significant difference
P8	16.67	117%	0.093	1.093 ± 0.021	0.973 ± 0.021	0.008	Significant difference
P9	16.67	114%	0.055	0.922 ± 0.008	0.889 ± 0.056	0.495	No significant difference
P10	16.67	109%	<0.014	0.332 ± 0.203	n.c.	0.111	No significant difference
P11	16.67	117%	0.018	0.375 ± 0.031	0.181 ± 0.107	0.097	No significant difference
P12	16.67	102%	0.023	0.476 ± 0.097	0.321 ± 0.114	0.218	No significant difference
P13	16.67	112%	n.d.	0.007 ± 0.016	n.c.	0.455	No significant difference
P14	16.67	85%	0.115	0.375 ± 0.070	0.985 ± 0.013	<0.001	Significant difference
P15	1.85	107%	0.014	1.048 ± 0.038	0.092 ± 0.082	<0.001	Significant difference

n.d. not detected; n.c. not calculated, for samples with detected pheophorbide A level in range of a protective effect below 10%, p values refer to comparison against the predefined assay variability interval of 0 ± 0.1; * measured in technical duplicate on the same assay plate; predicted effects were calculated from the pheophorbide A concentration–response model established in three independent experiments (n = 3); significance level p < 0.05.

In the following, different examples were selected to illustrate the various cases that may arise when applying the proposed method.

Case I: Pheophorbide A was detected in 12 out of the 15 crude plant extracts analyzed, with the exceptions being *Centaurium erythraea* (P3) and *Verbascum densiflorum* (P13), in which no pheophorbide A could be identified (Table 2). However, relying solely on LC-PDA or LC-MS data for extract selection would be insufficient in this case: the two extracts lacking pheophorbide A exhibited no protective effect, whereas the remaining extracts would have been excluded based solely on the presence of pheophorbide A – despite the potential to contain additional active constituents.

Case II: The protective effect observed for *Cichorium intybus* (P4; c = 100 µg/mL, 0.139 ± 0.004) and *Veronica officinalis* (P14, c = 16.67 µg/mL, 0.375 ± 0.070) were lower than expected due to calculation of the predicted effect using pheophorbide A content (Table 2). This discrepancy can be attributed to a reduction of cell viability (Table 2), which was also assessed as control in the screening assay used with solely extract and no viral treatment. At the tested concentration, *Veronica officinalis* extract (P14; c = 16.67 µg/mL) resulted in only 85% cell viability compared to untreated controls, while *Cichorium intybus* extract (P4; c = 100 µg/mL) showed a cell viability of just 69% (Table 2). A concentration- and time-dependent reduction in cell viability was also seen in breast cancer SKBR3 cell line for *C. intybus* extracts [28]. Reducing effects on cell viability may be attenuated in later stages of the investigation of these plants, thereby allowing a protective effect to become apparent. However, since the presented strategy is designed for prioritization in screening, pragmatic cut-off values are required, in this case based on cell viability. Accordingly, these plants were not further investigated in this screening study aiming to identify additional protective compounds.

Case III: The protective effect of *Melissa officinalis* (P6; c = 50 µg/mL, p = 0.087), *Thymus vulgaris* (P11; c = 16.67 µg/mL, p = 0.097), *Urtica dioica* (P12; c = 16.67 µg/mL, p = 0.218), and *Sambucus nigra* (P9; 16.67 µg/mL, p = 0.495) did not significantly deviate from the pheophorbide A-based predicted protective effect (Table 2). Accordingly, the observed effects may be consistent with the contribution of pheophorbide A to the antiviral effect of these crude extracts.

Case IV: A difference between the predicted and the measured protective effect (Fig 2A) suggested the possible presence of additional antiviral constituents other than pheophorbide A. Based on the concentration of the extracts exhibiting a protective effect of more than 80% and a difference between the predicted and measured protective effect (Table 2), *Artemisia annua* (P1, p = 0.003), *Artemisia vulgaris* (P2, p = 0.006), *Geranium robertianum* (P5, p < 0.001), *Primula veris* (P7, p = 0.016), *Salix alba* (P8, p = 0.008), and *Viola odorata* (P15, p < 0.001) were selected for further investigations.

*Artemisia annua* (P1) showed a protective effect measured that is higher than 100%, which is likely attributed to increased cell viability and/or metabolic activity relative to untreated controls, since the extract control measured on the same plate without virus treatment also demonstrated a cell viability of 122% at a concentration of 5.56 µg/mL (Table 2). Values exceeding 100% of cell viability have also been reported for *Artemisia*-derived compounds in other cell-based assays [29].

Accordingly, the crude extracts of promising plants were subjected to further prefractionation using liquid-liquid extraction, resulting in an organic and an aqueous prefraction.

### Prioritization of prefractions

The subsequently obtained organic and aqueous prefractions were investigated using LC-PDA, the pheophorbide A content was determined and the predicted protective effect was calculated. (Table 3 and Table 4). Since almost all organic prefractions contained pheophorbide A (Table 3), all would have been excluded if the mere presence of pheophorbide A would have been used as the exclusion criterion. However, the comparison between the protective effect measured and the predicted protective effect (Table 3, Fig 2B) indicated that the effect of the organic prefractions of *Artemisia annua* (P1, p < 0.001), *Artemisia vulgaris* (P2, p = 0.001), *Geranium robertianum* (P5, p = 0.006), *Primula veris* (P7, p = 0.031), and *Salix alba* (P8, p < 0.001) appeared to be at least partially independent of the pheophorbide A content.

**Table 3:** Concentration of organic prefraction applied in the pretreatment assay, corresponding cell viability, pheophorbide A content and comparison of measured and predicted effect.

Plant code	Concen-tration (µg/mL)	Cell viability (% of untreated control)	Pheophorbide A (µg/mL)	Measured effect (mean±SD)*	Predicted effect (mean±SD, n = 3)	p value	Result of comparison measured and predicted protective effect
P1	1.85	84%	<0.014	0.992 ± 0.013	n.c.	<0.001	Significant difference
P2	1.85	112%	0.018	1.130 ± 0.026	0.190 ± 0.108	0.001	Significant difference
P5	5.56	98%	0.015	0.878 ± 0.162	0.113 ± 0.091	0.006	Significant difference
P7	5.56	117%	0.064	1.163 ± 0.100	0.927 ± 0.042	0.031	Significant difference
P8	5.56	101%	<0.014	1.130 ± 0.026	n.c.	<0.001	Significant difference
P15	5.56	106%	0.119	0.923 ± 0.024	0.987 ± 0.012	0.424	No significant difference

n.c. not calculated, for samples with detected pheophorbide A level in range of a protective effect below 10%, p values refer to comparison against the predefined assay variability interval of 0 ± 0.1; * measured in technical duplicate on the same assay plate; predicted effects were calculated from the pheophorbide A concentration–response model established in three independent experiments (n = 3); significance level p < 0.05.

**Table 4:** Concentration of aqueous prefraction applied in the pretreatment assay, corresponding cell viability, pheophorbide A content and comparison of measured and predicted effect.

Plant code	Concen-tration (µg/mL)	Cell viability (% of untreated control)	Pheo-phorbide A (µg/mL)	Measured effect (mean±SD)*	Predicted effect (mean±SD, n = 3)	p value	Result of comparison measured and predicted protective effect
P1	**16.67**	109%	<0.014	0.128 ± 0.075	n.c.	0.226	No significant difference
P2	**16.67**	97%	<0.014	0.163 ± 0.024	n.c.	0.120	No significant difference
P5	**16.67**	99%	<0.014	0.129 ± 0.162	n.c.	0.339	No significant difference
P7	**16.67**	106%	<0.014	−0.092 ± 0.022	n.c.	0.310	No significant difference
P8	**16.67**	97%	<0.014	−0.079 ± 0.014	n.c.	0.370	No significant difference
P15	**16.67**	96%	<0.014	−0.098 ± 0.121	n.c.	0.390	No significant difference

n.c. not calculated, for samples with detected pheophorbide A level in range of a protective effect below 10%, p values refer to comparison against the predefined assay variability interval of 0 ± 0.1; * measured in technical duplicate on the same assay plate; predicted effects were calculated from the pheophorbide A concentration–response model established in three independent experiments (n = 3); significance level p < 0.05.

The observed effect of *Viola odorata* (P15, p = 0.424) (Fig 2B) did not significantly deviate from the predicted protective effect and may therefore be consistent with the contribution of pheophorbide A. Several explanations may account for this discrepancy from the results of crude extract, including A) as this is a screening assay, the antiviral assay was performed in technical duplicate limiting the ability to reliably identify potential measurement artefacts, such as unintended light exposure [17]. B) The compounds responsible for the observed effect may have been degraded or modified in the liquid-liquid extraction. C) The compounds separated in the prefractionation may have a synergistic effect in crude extracts, which could explain the reduced effect observed after fractionation. The topic of the synergistic effects of plant extracts is of great current interest [30], and research into the antiviral activity is therefore of considerable importance, however was beyond the scope of the presented study. D) In general, compounds that interfere with the luciferase readout can be separated during liquid–liquid extraction [26,31], but may then still appear as effective in the aqueous fraction due to the partitioning behavior. Accordingly, also the effect of the aqueous prefractions has to be taken into account for interpretation of inconsistent findings of crude-extract and organic prefraction.

The aqueous prefractions presented a distinct picture (Fig 2C, Table 4). A comparison of the predicted protective effect with the measured effect of the aqueous prefractions, revealed that no measured effect was observed for *Viola odorata* (P15), *Salix alba* (P8), and *Primula veris* (P7). This finding aligns with the results of a previous study that examined the antiviral activity of different *Primula* extracts against the H1N1 virus, demonstrating that the water extract was not active [32]. Low protective effects were observed for the two *Artemisia species* (P1 0.128 ± 0.075 and P2 0.163 ± 0.024), as well as for *Geranium robertianum* (P5 0.129 ± 0.162). However, these effects did not differ significantly from the predefined assay variability and therefore do not support prioritization within the present screening workflow. At most, the results may be regarded as exploratory indications for future studies of polar constituents, some of which have previously been reported to exhibit antiviral activity [33]. The low or absent activity in the aqueous fractions may also partly reflect methodological limitations, including assay sensitivity, solubility constraints, or potential instability during freeze-drying. A comprehensive investigation of these aspects is beyond the scope of the present study, which is centered on the methodological leveling-out approach.

### Prioritization of fractions derived from prefractions

The most promising organic prefractions were further fractionated using a step gradient with rising polarity (*n*-hexane (100% to 0%) and ethyl acetate (0% to 100%)) to yield 12 fractions. A higher number of fractions is generally associated with the production of purer samples and an increased probability of identifying minor bioactive compounds. However, this benefit must be balanced with the increasing costs [34] and experimental effort involved, particularly under restrictive conditions such as those in biosafety level 3 (BSL-3) laboratories.

The results of *Primula veris (P7)* are discussed in this context. The protective effects of all fractions were analyzed using the pretreatment assay, while the pheophorbide A content was evaluated through LC-PDA (Fig 2D, Table 5).

**Table 5:** Comparison of measured and predicted effect of fractions of P7.

P7	Cell viability (% of untreated control)	Measured effect (mean±SD)*	Predicted effect (mean±SD, n = 3)	p value	Result of comparison measured and predicted protective effect
Fraction 1	112%	−0.001 ± 0.016	n.c.	0.957	No significant difference
Fraction 2	115%	0.021 ± 0.023	n.c.	0.079	No significant difference
Fraction 3	115%	0.326 ± 0.178	n.c.	0.039	Significant difference
Fraction 4	109%	0.022 ± 0.051	n.c.	0.799	No significant difference
Fraction 5	124%	1.070 ± 0.079	0.478 ± 0.113	0.008	Significant difference
Fraction 6	99%	0.961 ± 0.030	0.960 ± 0.028	0.960	No significant difference
Fraction 7	92%	0.784 ± 0.018	0.575 ± 0.111	0.086	No significant difference
Fraction 8	116%	0.080 ± 0.011	n.c.	0.364	No significant difference
Fraction 9	123%	0.092 ± 0.167	n.c.	0.484	No significant difference
Fraction 10	114%	0.114 ± 0.043	n.c.	0.241	No significant difference
Fraction 11	130%	0.267 ± 0.091	n.c.	0.026	Significant difference
Fraction 12	116%	0.060 ± 0.044	n.c.	0.256	No significant difference

n.c. not calculated, for samples with detected pheophorbide A level in range of a protective effect below 10%, p values refer to comparison against the predefined assay variability interval of 0 ± 0.1; * measured in technical duplicate on the same assay plate; predicted effects were calculated from the pheophorbide A concentration–response model established in three independent experiments (n = 3); significance level p < 0.05.

The mean of the protective effect determined using the pretreatment assays is represented as bars±SD, while the mean of protective effect predicted by pheophorbide A content is displayed using green lines±SD (Fig 2D). Pheophorbide A is predominantly present in fractions 5–7 (Fig 2D black squares, Table 5). In conventional bioactivity-guided screening approaches, these fractions would likely be selected for further investigations due to high biological activity. However, the measured effect of fraction 6 (p = 0.960) and fraction 7 (p = 0.086) do not differ significantly from predicted effects and therefore provided no indication of additional effects. However, comparison of the measured and predicted protective effects revealed that fractions 3 (p = 0.039), fraction 5 (p = 0.008), and fraction 11 (p = 0.026) significantly deviated from the pheophorbide A-based prediction (Table 5).

Particularly noteworthy is fraction 5, which would typically be excluded in a conventional approach due to the presence of pheophorbide A. However, fraction 5 exhibited a substantially stronger measured protective effect (1.070 ± 0.079) compared to the predicted effect (0.478 ± 0.113), suggesting the presence of additional bioactive constituents besides pheophorbide A (p = 0.008).

Likewise, fractions 3 (measured effect 0.326 ± 0.178) and 11 (measured effect 0.267 ± 0.091) emerged as promising candidates for further phytochemical investigation, due to the comparatively high measured effects despite low predicted effect.

To estimate at which point an effect independent of pheophorbide A becomes detectable in the crude extract, the relative mass contributions of the fractions showing significant differences between the measured and predicted effects were calculated. The mass of fraction 3 and fraction 11 yields a portion of 11.9%-mass of the crude extract, which seems to be sufficient to be recognized in the approach used, as a promising plant extract for further investigations (Table 6). The mass percentage of the fractions of the other plant species, which demonstrated a protective effect that is not solely dependent on pheophorbide A content, is in the range of 4.3–15.2%-mass of crude extract (S1 Table). Within this 4.3%, the effects of possible additional components are likely masked by the dominant activity of pheophorbide A.

**Table 6:** Mass% of organic prefraction of the crude extract (CE) and masses of the 12 fractions obtained from gradient with rising polarity.

Plant code	CE(%)	F1(mg)	F2(mg)	F3(mg)	F4(mg)	F5(mg)	F6(mg)	F7(mg)	F8(mg)	F9(mg)	F10(mg)	F11(mg)	F12(mg)
**P7**	49.2	4.02	10.00	5.75	3.30	4.77	9.07	13.70	11.01	6.70	4.41	9.49	12.12

### Identification of candidate compounds

**Fig 3:**
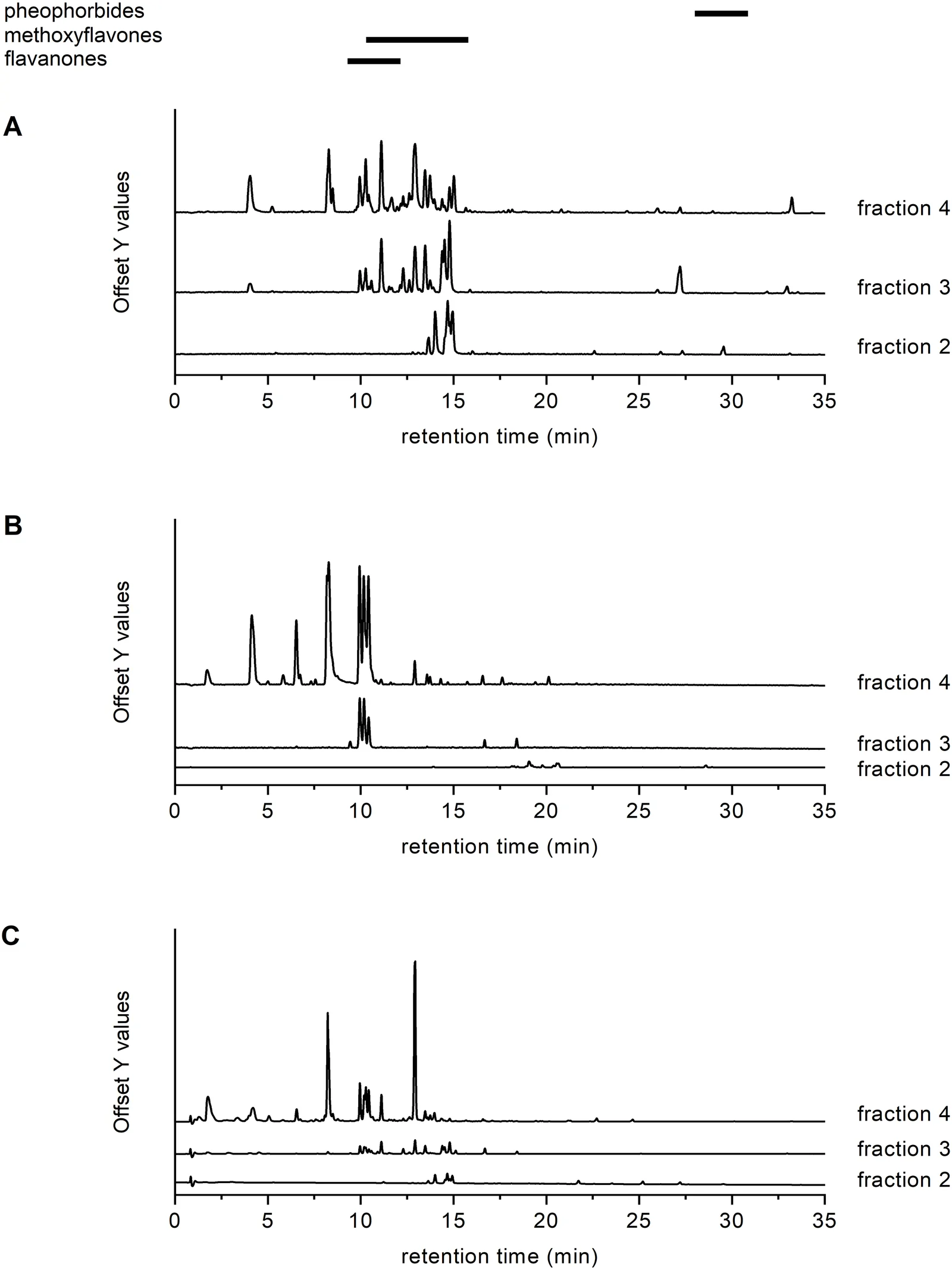
Chromatograms of *Primula veris* (P7) fraction 2, fraction 3 and fraction 4. A) Base peak chromatogram (BPC) positive ionization mode; B) BPC negative ionization mode; and C) absorption at λ = 200 nm.

Based on the presented method, crude extracts and prefractions were prioritized for further investigation, and the method was evaluated with regard to the content of bioactive compounds. Following the selection of fractions whose effects were either not attributable or only partially attributable to pheophorbide A (Fig 2D, Table 5), the components in fraction 3 of *Primula veris (P7)* were further investigated. Therefore, an orthogonal approach (bioactivity compared with semiquantitative feature detection) was employed to identify possible compounds in fraction 3 of *Primula veris (P7)* using untargeted analytical evaluation by recording LC-HRESIMS metabolite profiles (Fig 3). The data obtained from the LC-HRESIMS analysis of the different fractions under investigation and the adjacent fractions were compared pairwise in a semiquantitative manner using the XCMS web interface [35]. In contrast to the quantitative analysis of pheophorbide A, features were compared using feature areas (semi-quantitative, S6-S9 Tables). The resulting features were refined according to the following criteria: A) the feature quantity is less in inactive fractions, B) the feature does not belong to fragments, C) the feature does not belong to isotopes, D) the feature abundance is more than 1.000.000, and E) the abundance of the feature is more than 1.5 fold in the active fraction than in the less active fractions.

A comparison of the total ion chromatograms (TICs) of fractions 3 and 2 of negative ionization mode (Fig 3B (BPC)) yielded 80 distinct features (S6 Table). Subsequent refinement of these results, in conjunction with the outcomes of the comparison between fractions 3 and 4 (S7 Table), led to the reduction of the feature count to three, which are summarized in Table 7.

**Table 7:** LC-PDA-HRESIMS data of compounds more abundant in fraction 3 than fractions 2 and 4 using negative ionization mode.

Feature	Retention time (min)	Ion	m/z measured	m/z predicted	MS/MS	λ_max_(nm)	Proposed molecular formula
**1**	9.54	[M-H]^–^	271.0610	271.0612	177, 151, 119	290	C_15_H_12_O_5_
**2**	16.77	[M-H]^–^	423.1597	423.1581	317, 299, 210	281	C_28_H_24_O_4_
**3**	18.51	[M-H]^–^	425.1757	425.1758	317, 211	275	C_28_H_26_O_4_

As illustrated in Table 7, feature 1 shows an absorption maximum of λ = 290 nm, which is characteristic of the group of flavanones. Together with the observed [M − H]^-^ ion at m/z 271, these data were consistent with feature 1 being naringenin. This assignment was confirmed by matching retention time and LC-HRESIMS data with an authentic reference standard (S2 Fig).

Feature 2 and 3 (Table 7) show related fragmentation patterns and similar absorption maxima. Accordingly, both should be classified as belonging to the same compound class. A search in Lotus Database [36] revealed the presence of a possible compound, Riccardin C. Riccardin C has previously been identified in *Primula veris* [32,37] and the absorption maxima of λ = 281 nm and 275 nm are consistent with those reported in literature [37].

A comparison of the TICS of fraction 3 and fraction 2 of positive ionization mode (Fig 3A (BPC)) yielded 46 features that were more abundant in fraction 3 (S8 Table). These features were refined by comparison with features of fraction 4 (S9 Table) resulting in a reduction to 15 features, which were further refined by abundance and isotope signals, resulting in a final set of 9 features for further analysis (Table 8).

**Table 8:** LC-PDA-HRESIMS data of compounds more abundant in fraction 3 than in fractions 2 and 4 using positive ionization mode.

Feature	Retention time (min)	Ion	m/z measured	m/z predicted	λ_max_ (nm)	Proposed molecular formula
1	10.68	[M+H]^+^	239.0683	239.0703	245, 309	C_15_H_10_O_3_
2	12.93	[M+H]^+^	557.1815		224, 266	
3	13.06	[M+H]^+^	343.1147	343.1176	230, 310, 328	C_19_H_18_O_6_
4	14.47	[M+H]^+^	297.0733	297.0758	236, 309	C_17_H_12_O_5_
5	14.60	[M+H]^+^	253.0845	253.0859	236, 309	C_16_H_12_O_3_
6	14.86	[M+H]^+^	283.0949	283.0965	244, 294, 350	C_17_H_14_O_4_
7	27.31	[M+H]^+^	425.2885			
8	32.00	[M+H]^+^	607.2902		410, 665	
9	33.07	[M+H]^+^	621.3053	621.3071	407, 665	C_37_H_40_N_4_O_5_

As illustrated in Table 8, feature 6 shows absorption maxima at λ = 244, 294, 350 nm and can be attributed to a dimethoxyflavone based on HRESIMS data, which was previously identified in *Primula* [38]. Accordingly, HRESIMS analysis indicates feature 5 (Table 8) to possibly be a methoxyflavone [32], feature 4 (Table 8) to be a flavone with an ether bridge on the B-ring such as 3’-methoxy-4,’5’-methylenedioxyflavone [38], and feature 1 (Table 8) to be a hydroxyflavone, already identified in *Primula* [32]. Additionally, feature 3 (Table 8) may also be classified within this compound group, based on the observed absorption maxima and the predicted sum formula, which aligns with a tetramethoxyflavone [32]. The group of polymethoxylated flavones is known constituents of *Primula* species [32,38].

Feature 8 and 9 (Table 8) show absorption maxima at λ = 410 and 665 nm. This observation leads to the conclusion that these are chlorophyll breakdown products such as pheophorbide A ethyl ester [12].

The comparison of fractions 2, 3 and 4 (Table 7, Table 8, Fig 3) revealed flavanones, and methoxyflavones in addition to pheophorbides as possible candidates contributing to the antiviral effect of *Primula veris* (P7). Compound annotation ranged from tentative class assignment to confirmation by comparison with authentic reference standards. Compounds confirmed by authentic reference standards were prioritized for subsequent antiviral evaluation, whereas the remaining candidate compounds warrant further phytochemical and pharmacological investigation to clarify the individual contributions to the observed antiviral activity.

### Confirmation of protective effect of identified compounds

**Fig 4:**
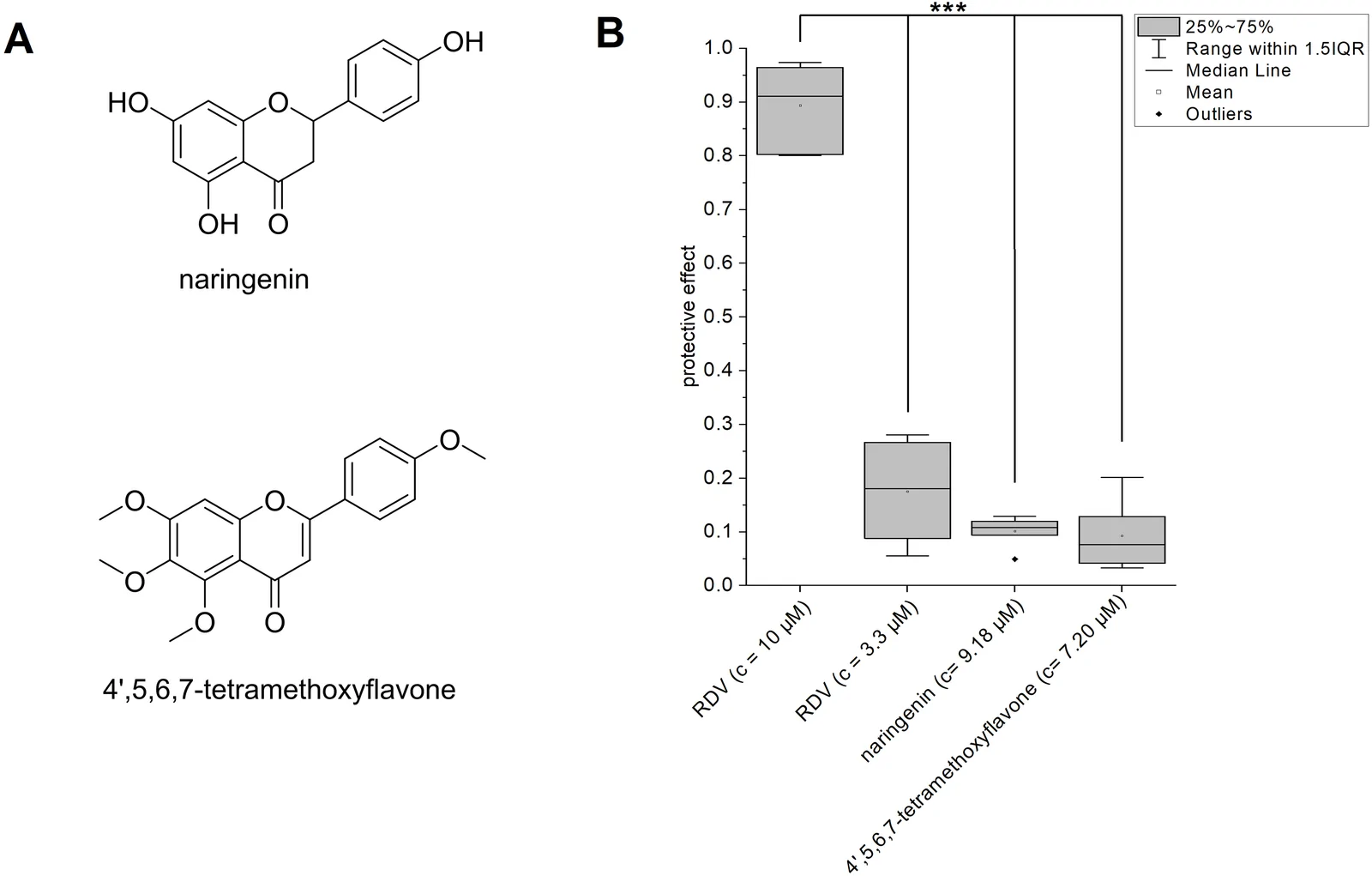
A) Chemical structures of naringenin and 4′,5,6,7-tetramethoxyflavone, B) Protective effects of identified compounds compared with the positive control Remdesivir (RDV) (n = 3, ***p < 0.001).

Accordingly, to complete the study presented, the protective effects of the identified and commercially available compounds naringenin and 4’,5,6,7-tetramethoxyflavone (Fig 4A) were determined in the *in vitro* virus pretreatment assay. For Remdesivir (RDV), which serves as a positive control, a protective effect of 0.894 ± 0.078 was determined using the pretreatment assay at a concentration of 10 µM, which corresponds to 6.02 µg/mL and a protective effect of 0.175 ± 0.0945 at a concentration of 2.00 µg/mL. Naringenin and 4’,5,6,7-tetramethoxyflavone were screened at a concentration of 2.5 µg/mL. At this concentration, none of the compounds tested showed a significant impact on cell viability using virus-untreated Vero E6 cells.

Naringenin (Fig 4A) demonstrated a protective effect of 0.101 ± 0.028 (n = 3, mean±SD). A review of the recent literature on the effects of naringenin in the context of SARS-CoV-2 reveals a number of studies that summarize the various effects and the impact of naringenin [39–41]. Methoxylated flavonoids exist in a multitude of isomeric forms, rendering identification and bioactivity without isolation challenging. Therefore, the commercially available 4’,5,6,7-tetramethoxyflavone (Fig 4A) was used as a representative compound of tetramethoxylated flavones, although this compound has not been directly identified in fraction 3 of *Primula veris* (S3 Fig). The screening of 4’,5,6,7-tetramethoxyflavone (Fig 4B) demonstrated a protective effect of 0.092 ± 0.063 (n = 3, mean±SD). 4’,5,6,7-Tetramethoxyflavone is also known as tetramethylscutellarein, a member of the subgroup of polymethoxylated flavones, which display a wide range of biological activities, including antiviral properties [42].

The protective effects observed for naringenin (p = 0.50) and 4’,5,6,7-tetramethoxyflavone (p = 0.33) did not differ significantly from that of Remdesivir (RDV) at a concentration of 3.3 µM using the pretreatment assay. Both compounds have previously been identified as antiviral in other studies [39–42] and demonstrated an effect in the *in vitro* pretreatment assay, thereby supporting the suitability of the presented method for prioritizing extracts and candidate compounds in screening workflows for subsequent in-depth investigations.

## Conclusion

The identification of antiviral compounds in plants and plant extracts is a challenging process due to the vast number of plant varieties, the diverse geographical influences on phytochemical composition, the multitude of extraction methods, and multicomponent composition of plant extracts in dependence of extraction conditions. In classical approaches, such as bioactivity-guided fractionation, this complexity translates into extensive experimental effort and iterative testing steps. Moreover, when targeting antiviral activity, these procedures can be particularly demanding in high-containment laboratory settings, where handling infectious agents significantly increases logistical constraints, workload, and biosafety requirements. The strategy presented enables the prediction of contribution of a known compound to observed effects, in this case pheophorbide A, and supports the prioritization of extracts and (pre)fractions where the observed effect is not primarily driven by the known compound. By leveling out such dominant influences, the approach aids in identifying candidates for further investigation that might otherwise remain masked. Although demonstrated here for pheophorbide A in an antiviral pretreatment assay, the presented strategy may be adaptable to other screening workflows in which dominant known constituents interfere with extract prioritization.

## Materials and methods

### Plant material and compounds

*Primula veris* L., inflorescence including peduncle, was harvested in 2022 from a field of commercial cultivation and supplier near Straubing (Germany) (Gäuboden Kräuter GmbH & Co. KG). The other European Medicinal Plants were purchased from Alfred Galke GmbH (Bad Grund, Germany) or Dixa AG (St. Gallen, Switzerland) and Médiplant (Conthey, Switzerland). As the herbal raw materials were obtained from commercial suppliers or cultivated sources and not collected from the wild, no voucher specimens were prepared. Instead, retained samples of the comminuted plant material were deposited in our institutes for future reference. The purchased dried plant material was ground and sieved (<1 mm) and stored in brown glass vessels at a temperature of 4°C in the dark until further processing. Hydroalcoholic extracts were prepared using 40 g of plant material and 468 mL 80% v/v analytical grade ethanol (VWR Chemicals, Darmstadt, Germany). The mixture was stirred at room temperature for 2 hours in a closed 500 mL Erlenmeyer flask. The solution was filtered through qualitative pleated filter paper (VWR, Darmstadt, Germany) with high filtration speed and a particle retention of 12–15 µm. The filtered extract was evaporated under reduced pressure at 40°C until dryness. The extracts were stored at 4°C in the dark until further use. The reference compounds were purchased, naringenin (Merck KGaA, Darmstadt, Germany), 4’,5,6,7-tetramethoxyflavone (Merck KGaA, Darmstadt, Germany), and pheophorbide A (Cayman, Ellsworth, USA) and used without further purification.

### Prefractionation

Prefractionation of hydroalcoholic extracts was performed via liquid-liquid extraction by dissolving 500 mg of extract in 200 mL distilled water (pH 5) and 200 mL of analytical grade ethyl acetate (VWR Chemicals, Darmstadt, Germany). After phase separation, the organic layer was removed and the aqueous phase was exhaustively extracted with five additional portions of ethyl acetate (V = 50 mL). The combined organic phases were washed once with 100 mL of distilled water (pH 5) and the solvent was evaporated under reduced pressure at 40°C. The aqueous phase was frozen in liquid nitrogen and freeze dried using an Alpha 2–4-LD plus freeze dryer (Christ, Osterode, Germany). The dried extracts were stored at 4°C in the dark until further use.

### Fractionation

The organic prefractions were loaded on silica gel (60–200 µm; 1:10) (VWR, Darmstadt, Germany) and fractionated using solvent A (analytical grade *n*-hexane, VWR Chemicals, Germany) and solvent B (analytical grade ethyl acetate, VWR chemicals, Darmstadt, Germany) on a Puriflash 4250 system (Interchim, Montlucon, France). Each step was performed using 75 mL solvent at a flow rate of 15 mL/min. The final step consisted of 225 mL of 10% methanol (analytical grade, VWR chemicals, Darmstadt, Germany) in ethyl acetate with the same flow rate.

### LC-PDA-HRESIMS-analysis

The LC-PDA-HRESIMS analysis was performed using a Shimadzu system, comprising two LC-20AD pumps, an autosampler, a column oven, PDA detector and the IT TOF. A reversed-phase Phenomenex Kinetex C_18_ column (2.1 × 100 mm, 2.6 µm) with solvents A (water + 0.1% formic acid) and B (acetonitrile + 0.1% formic acid) was employed. The flow rate was maintained at 0.4 mL/min at 30°C, with a gradient from 15% to 95% B over 35 minutes, followed by a 15-minute hold at 95%. The HRESIMS was recorded in both negative and positive ionization modes. MS¹ (m/z 100–1000) and MS² (m/z 50–1000) were acquired with ion accumulation times of 10 ms and 30 ms, respectively. Extracts (10 mg/mL) and fractions (5 mg/mL) were dissolved in methanol, and 5 µL were injected for analysis. For data analysis LCMS solution version 3.80.410 (Shimadzu) and the implemented Formula Predictor version 1.12 were used. Furthermore, the chemoinformatic supported evaluation was performed using XCMS. To compare the data, mzXML or cdf files were uploaded to the XCMS web interphase (https://xcmsonline.scripps.edu). As parameters for feature detection centWave with m/z deviation of 20 ppm, minimum peak width of 5 seconds, maximum peak width 40 s, and a minimum difference in m/z of 0.01 and for retention time correction the obiwarp method were chosen.

### Protective effect

The cell culture procedure and the pretreatment assay were performed as reported in Vahekeni *et al.* [24] (see also S1 File) and conducted at the Spiez Laboratory (Switzerland). Briefly, plant extracts were resuspended in DMSO to a concentration of 25 mg/mL and diluted to 400 μg/mL, 200 μg/mL, 66.67 μg/mL, 22.22 μg/mL, and 7.41 μg/mL in 2%-FCS-MEM. Fifty microliters of each concentration were added in duplicate to the upper half of a 96-well plate for antiviral testing and to the lower half for toxicity testing. Virus controls (infected, untreated) and cell controls (untreated, uninfected) were included, along with Remdesivir and DMSO controls. Plates were transferred to the BSL-3 lab, and 100 PFU of SARS-CoV-2 (2019-nCoV/IDF0372/2020) in 50 µL culture medium was added to the upper wells, with 50 µL culture medium added to the lower wells and cell controls. After 1-hour incubation in the dark at 37°C and 5% CO_2_, 100 µL of Vero E6 cells (2 x 10^5^ cells/mL) was added resulting in a final concentration of extracts of 100 μg/mL, 50 μg/mL, 16.67 μg/mL, 5.56 μg/mL and 1.85 μg/mL and of DMSO to less than 0.8%. After 72 hours incubation in the dark, cell viability was measured using the CellTiter-Glo® assay on the GloMax instrument (Promega).

### Software

The software package OriginPro 2021b (64-bit) SR2 was employed for the purposes of data analysis, statistical analysis and graph preparation.

### Data and statistical analysis

Samples were assessed for effects on host cell viability and for the protective effects against virus-induced reduction of cell viability in a virus pretreatment assay. Crude extracts and (pre)fractions were evaluated using technical duplicates measured on the same assay plate to determine the experimentally measured protective effect. The protective effect measured was calculated as follows:


$$\begin{array}{c} \;protective\;effect=\frac{{luminescence}_{\;compound\;treated\;cells}-{luminescence}_{virus\;treated\;cells}}{{luminescence}_{untreated\;cells}-{luminescence}_{virus\;treated\;cells}} \end{array}$$

The predicted effect was calculated via quantification of pheophorbide at a wavelength of 650 nm and using the concentration-response curves of pheophorbide A, which were recorded in three independent experiments. Differences between groups were evaluated by one-way ANOVA followed by Bonferroni post hoc tests (significance level of p < 0.05). Statistical comparisons therefore evaluated whether experimentally measured effects deviated from the pheophorbide A-based calculated effect. For samples with pheophorbide A concentrations below 0.014 µg/mL, corresponding to predicted protective effects below the IC₁₀ threshold, the experimentally measured protective effects were statistically evaluated against a predefined assay variability interval of 0 ± 0.1. This interval was derived from the variability observed in the assay controls.

Purchased reference compounds were tested using three independent biological replicates to confirm the presence of a protective effect under the assay conditions. Differences between groups were evaluated by one-way ANOVA followed by Bonferroni post hoc tests. Data are presented as box plots.

## Supporting information

S1 Fig Concentration-effect curve of pheophorbide A tested in the antiviral pretreatment assay. (TIF)

S2 Fig XIC of m/z 271.06 in LC-HRESIMS chromatogram of fraction 3 and naringenin. (TIF)

S3 Fig XIC of m/z 343.12 in LC-HRESIMS chromatogram of fraction 3 and 4’,5,6,7-tetramethoxyflavone. (TIF)

S1 File Step-by-step protocol for cell culture and the antiviral assay. (PDF)

S1 Table Masses of the twelve fractions of the prioritized plants including % of active fractions with effect not solely dependent on pheophorbide A content. (PDF)

S2 Table Luminescence values used for calculation of cell viability and protective effect of crude extracts. (PDF)

S3 Table Luminescence values used for calculation of cell viability and protective effect of organic prefractions. (PDF)

S4 Table Luminescence values used for calculation of cell viability and protective effect of aqueous prefractions. (PDF)

S5 Table Luminescence values used for calculation of cell viability and protective effect of fractions. (PDF)

S6 Table Feature table received from comparison of fraction 3 and fraction 2 using XCMS measured in negative ionization mode. (PDF)

S7 Table Feature table received from comparison of fraction 3 and fraction 4 using XCMS measured in negative ionization mode. (PDF)

S8 Table Feature table received from comparison of fraction 3 and fraction 2 using XCMS measured in positive ionization mode. (PDF)

S9 Table Feature table received from comparison of fraction 3 and fraction 4 using XCMS measured in positive ionization mode. (PDF)
