# Supplementary material for: A bioactivity-leveling approach for component-specific evaluation in natural product extracts: Optimizing SARS-CoV-2 antiviral screening by controlling for pheophorbide A
Source: PLoS One. 2026 Aug 24;21(8):e0355466. doi: 10.1371/journal.pone.0355466 (PMC13502696; doi:10.1371/journal.pone.0355466)
Supplement: Supplementary file 1 [file pone.0355466.s004.pdf]

Step by Step protocol of Cell culture and Antiviral Assay

Cells and pathogenic viruses

Vero E6/TMPRSS2 cells were passaged in Dulbecco's modified Eagle medium (DMEM) containing 10% fetal calf serum (FCS), 100 U/mL of penicillin, 100 µg/mL of streptomycin, and 1 mg/mL geneticin (all from Bioswisstec). SARS-CoV-2 (2019-nCoV/IDF0372/2020) clade 19A was obtained from the National Reference Centre for Respiratory Viruses hosted at the Institut Pasteur (Paris, France), propagated in Vero E6/TMPRSS2 cells in DMEM containing 2% FCS, supplements (2%-FCS-DMEM), and 1 mg/mL geneticin at 37 °C, >85% humidity, and 5% CO_2. The viral titer was determined using a standard plaque assay by incubating tenfold serial dilutions of the virus for 1 h at 37 °C on a confluent 24well plate with Vero E6/TMPRSS2 cells. Then, the inoculum was removed, and 1 mL of overlay medium (20 mL DMEM, 5 mL FCS, 100 U/mL of penicillin, 100 µg/mL of streptomycin, and 25 mL of Avicel rc581) was added. After 3 days of incubation at 37 °C, the overlay was removed, and the plates were stained with crystal violet. Due to the clearer readout, Vero E6 cells in suspension were used for the antiviral assays. Vero E6 cells were passaged in minimal essential medium (MEM) containing 10% FCS and supplements (2 mM L-glutamine, non-essential amino acids, 100 U/mL of penicillin, 100 µg/mL of streptomycin, and 1.5 g/ L sodium bicarbonate, all from Bioswisstec) at 37 °C, >85% humidity, and 5% CO_2.

Antiviral Assay

The protective activity of the extracts was assessed in a virus preincubation experiment. The plant extracts were resuspended in DMSO to a concentration of 25 mg/mL and diluted to concentrations of 400 µg/mL, 200 µg/mL, 66.6 µg/mL, 22.2 µg/mL, and 7.4 µg/mL in MEM containing 2% FCS and supplements (2 mM L-glutamine, non-essential amino acids, 100 U/mL of penicillin, 100 µg/mL of streptomycin, and 1.5 g/L sodium bicarbonate, all from Bioswisstec) (2%-FCS-MEM). The plant extracts, 50 µL of each concentration, were distributed in duplicates in the upper half of a flat-bottom, 96well plate (TPP, Trasadingen, Switzerland) to assess antiviral activity, and 50 µL of the same concentrations of the plant extracts were distributed in duplicate in the lower half of the 96well plate to assess cell viability or toxicity of the extracts. On each plate, 10 wells were used to determine the maximum viral cytopathogenicity (virus control VC) of infected but untreated cells, and 10 wells were used to determine the maximum cell viability (cell control CC) of uninfected and untreated cells. Further controls included serial dilutions of remdesivir as a positive control and DMSO at the same concentration as the extracts to control for the effects of the diluent. The plates were transferred to the BSL-3 laboratory, and 100 PFU of SARS-CoV2 virus (2019-nCoV/IDF0372/2020) in 50 µL culture medium (2%FCS-DMEM) was added to the upper half of the plate and to the virus control (VC) wells, while in the lower half of the 96-well plate, 50 µL of culture medium (2%-FCS-MEM) was added to the cell control wells. The plates were then incubated for 1 h at 37 °C and 5% CO_2, and 100 µL of Vero E6 cell suspension ($2 \times 10e^5$cells/mL) in cell culture medium (2%-FCS-MEM) was added to each well, leading to a final concentration of extracts of 100 µg/mL, 50 µg/mL, 16.7 µg/mL, 5.6 µg/ mL, and 1.9 µg/mL. The plates were then incubated for 72 h at 37 °C and 5% CO_2, after which cell viability was determined by the CellTiterGlo® Luminescent Cell Viability Assay (Promega, Madison, United States). Briefly, the CellTiter-Glo® suspension was prepared according to the manufacturer's protocol and mixed 1:1 with the cell culture medium (2%-FCS-MEM. The cell culture medium from the incubated plates was removed, and 200 µL of CellTiter-Glo®/medium mix was added to each well. The plates were shaken for 2 min at 400 rpm and incubated for 10 min at room temperature, and 100 µL of the CellTiter-Glo mix was transferred to a 96-well half-area white flatbottom plate (Corning® Costar® 3,693, Fisher Scientific). Luminescence, serving as a surrogate measure of host cell viability by determining cellular ATP levels, was quantified using the GloMax instrument (Promega, Madison, United States).
