# Supplementary material for: A bioactivity-leveling approach for component-specific evaluation in natural product extracts: Optimizing SARS-CoV-2 antiviral screening by controlling for pheophorbide A
Source: PLoS One. 2026 Aug 24;21(8):e0355466. doi: 10.1371/journal.pone.0355466 (PMC13502696; doi:10.1371/journal.pone.0355466)
Supplement: Supplementary file 2 [file pone.0355466.s005.pdf]

**Plant code**	**CE (%)**	**F1 (mg)**	**F2 (mg)**	**F3 (mg)**	**F4 (mg)**	**F5 (mg)**	**F6 (mg)**	**F7 (mg)**	**F8 (mg)**	**F9 (mg)**	**F10 (mg)**	**F11 (mg)**	**F12 (mg)**	**Active fraction (%)**
**P1**	92.2%	2.63	7.08	70.43	20.11	12.88	10.36	5.3	4.22	4.47	6.47	5.02	6.7	10.7%
**P2**	71.7%	3.9	14.95	1.42	4.84	0.14	3.98	3.45	5.38	2.74	8.57	5.8	16.09	6.3%
**P5**	90.9%	15.88	3.41	3.03	3.99	4.05	3.91	3.61	3.72	3.94	3.39	1.68	13.05	4.3%
**P8**	83.5%	4.49	1.97	3.01	4.63	2.95	1.68	1.81	2.21	1.8	1.64	3.77	11.97	15.2%
**P15**	84.3%	27.45	3.56	2.9	3.36	3.53	3.29	3.23	1.87	1.44	1.55	1.69	21.99	8.2%
