# Supplementary material for: A bioactivity-leveling approach for component-specific evaluation in natural product extracts: Optimizing SARS-CoV-2 antiviral screening by controlling for pheophorbide A
Source: PLoS One. 2026 Aug 24;21(8):e0355466. doi: 10.1371/journal.pone.0355466 (PMC13502696; doi:10.1371/journal.pone.0355466)
Supplement: Supplementary file 3 [file pone.0355466.s006.pdf]

crude extracts	Luminescence Value Virus-treated Cells		Luminescence Value Cell viability Control	
P1	6731000	6897000	6012000	6123000
P2	7731000	7052000	9209000	8983000
P3	2139000	1712000	6248000	5883000
P4	2677000	2680000	5167000	3957000
P5	6703000	6550000	6522000	6581000
P6	5893000	6153000	6406000	6214000
P7	6029000	5981000	6436000	5883000
P8	6697000	8193000	7527000	7908000
P9	7066000	7006000	7040000	7022000
P10	2537000	3845000	6293000	6356000
P11	3216000	3088000	5802000	5460000
P12	5224000	4811000	6731000	7035000
P13	2123000	2049000	5719000	5719000
P14	3228000	3269000	6132000	5506000
P15	6438000	6193000	6346000	6661000
