# Supplementary material for: A bioactivity-leveling approach for component-specific evaluation in natural product extracts: Optimizing SARS-CoV-2 antiviral screening by controlling for pheophorbide A
Source: PLoS One. 2026 Aug 24;21(8):e0355466. doi: 10.1371/journal.pone.0355466 (PMC13502696; doi:10.1371/journal.pone.0355466)
Supplement: Supplementary file 4 [file pone.0355466.s007.pdf]

organic prefraction	Luminescence Value Virus-treated Cells		Luminescence Value Cell viability Control	
P1	5074000	5016000	3621000	3746000
P2	8827000	9069000	9520000	9246000
P5	6685000	7740000	7536000	7665000
P7	6656000	5990000	6103000	6039000
P8	6165000	5671000	5701000	5771000
P15	3314000	2451000	7234000	6821000
