# Supplementary material for: A bioactivity-leveling approach for component-specific evaluation in natural product extracts: Optimizing SARS-CoV-2 antiviral screening by controlling for pheophorbide A
Source: PLoS One. 2026 Aug 24;21(8):e0355466. doi: 10.1371/journal.pone.0355466 (PMC13502696; doi:10.1371/journal.pone.0355466)
Supplement: Supplementary file 5 [file pone.0355466.s008.pdf]

aqueous prefraction	Luminescence Value Virus-treated Cells		Luminescence Value Cell viability Control	
P1	2277000	2600000	5357000	5552000
P2	2351000	2340000	5188000	5259000
P5	3766000	3651000	7687000	7618000
P7	1230000	935500	5103000	5265000
P8	1955000	1863000	5727000	5547000
P15	7959000	8128000	8522000	7914000
