# Supplementary material for: A bioactivity-leveling approach for component-specific evaluation in natural product extracts: Optimizing SARS-CoV-2 antiviral screening by controlling for pheophorbide A
Source: PLoS One. 2026 Aug 24;21(8):e0355466. doi: 10.1371/journal.pone.0355466 (PMC13502696; doi:10.1371/journal.pone.0355466)
Supplement: Supplementary file 6 [file pone.0355466.s009.pdf]

Fractions of P7	Luminescence Value Virus-treated Cells		Luminescence Value Cell viability Control	
F1	1978000	2080000	7542000	7459000
F2	2470000	2309000	7813000	7468000
F3	3278000	4525000	7889000	7558000
F4	2315000	1976000	7766000	7395000
F5	6946000	7473000	8931000	8697000
F6	7611000	7377000	7323000	6762000
F7	6440000	6585000	6783000	6350000
F8	2255000	2336000	7539000	7144000
F9	1728000	2900000	7383000	7338000
F10	2428000	2769000	7631000	7248000
F11	3090000	3801000	8663000	8282000
F12	2683000	2341000	8614000	8207000
