# Supplementary material for: A bioactivity-leveling approach for component-specific evaluation in natural product extracts: Optimizing SARS-CoV-2 antiviral screening by controlling for pheophorbide A
Source: PLoS One. 2026 Aug 24;21(8):e0355466. doi: 10.1371/journal.pone.0355466 (PMC13502696; doi:10.1371/journal.pone.0355466)
Supplement: Supplementary file 7 [file pone.0355466.s010.pdf]

featureidx	fold	updown	mzmed	rtmed	dataset1_mean	dataset2_mean
1	3710.238136	DOWN	269.0450448	10.05341667	67'857'271	0
2	3453.739449	DOWN	285.0401344	10.2984	63'166'116	0
3	2153.976773	DOWN	315.0508543	10.5484	39'394'502	0
4	649.6241403	DOWN	270.0482375	10.05341667	11'881'103	0
5	640.603944	DOWN	425.175743	18.51346667	11'716'131	0
6	566.9024067	DOWN	286.0429661	10.2734	10'368'189	0
7	504.6484691	DOWN	423.1597248	16.7734	9'229'615	0
8	474.5508598	DOWN	271.0610457	9.5384	8'679'153	0
9	467.6541392	UP	291.1958968	14.9393	0	8'553'018
10	385.1695417	DOWN	316.0542465	10.5484	7'044'441	0
11	361.8368727	UP	309.2075691	14.9393	0	6'617'705
12	290.893549	DOWN	299.0546129	10.37343333	5'320'209	0
13	225.5244733	DOWN	238.8913678	11.19843333	4'124'661	18'289
14	215.6182066	UP	293.2117027	16.66496667	0	3'943'484
15	204.406204	UP	511.2898131	33.2083	0	3'738'425
16	198.4269173	DOWN	265.1478987	19.48346667	3'629'069	0
17	186.9734725	UP	311.22249	16.66496667	0	3'419'594
18	186.816624	DOWN	238.8912442	9.870966667	3'726'142	19'945
19	184.6354857	DOWN	317.0671415	6.6534	3'376'835	0
20	184.2843397	DOWN	238.8926438	8.0634	3'370'412	0
21	183.9505838	DOWN	238.8919968	12.92341667	3'364'308	0
22	177.3902107	UP	327.2905713	23.44263333	0	3'244'324
23	173.3072155	DOWN	426.1782414	18.51346667	3'169'650	0
24	172.3981786	DOWN	345.0595213	10.39841667	3'153'024	0
25	164.8246825	DOWN	300.0281187	10.5484	3'014'511	0
26	164.1183721	DOWN	238.8921815	12.69841667	3'001'593	0
27	150.154318	UP	295.228535	15.72213333	0	2'746'202
28	146.2706871	UP	311.2226766	16.3613	0	2'675'173
29	142.5738673	DOWN	238.892547	7.765933333	2'999'409	21'038
30	134.2691095	DOWN	285.0416902	8.4234	2'455'674	0
31	124.6462478	UP	377.1961081	14.96046667	0	2'279'680
32	119.8835014	DOWN	345.0611393	8.0634	2'192'573	0
33	117.1574384	UP	291.1968444	15.12596667	0	2'142'715
34	110.933039	DOWN	238.8921569	7.4884	2'028'876	0
35	109.3591566	DOWN	301.0336973	8.3734	2'000'091	0
36	106.4056961	DOWN	260.8734335	14.49841667	1'946'075	0
37	106.0501971	DOWN	260.8737063	14.89841667	1'939'573	0
38	105.52123	DOWN	297.0771635	11.19843333	1'929'898	0
39	98.22105024	UP	293.1752824	9.6978	0	1'796'384
40	96.2969185	DOWN	238.8917653	13.89841667	20'870'175	216'727
41	91.62975461	DOWN	315.0522562	11.19843333	1'675'835	0
42	89.54049922	DOWN	329.0313172	10.8134	1'637'624	0
43	89.17377935	DOWN	238.8912409	12.36091667	3'365'192	37'737
44	86.58864808	DOWN	238.8914779	10.7634	7'246'597	83'690
45	82.96983117	DOWN	272.065442	9.5384	1'517'451	0

46	82.51304832	DOWN	238.8912526	11.8434	5'690'178	68'961
47	81.85060613	DOWN	313.0730332	13.32341667	1'496'982	0
48	78.16639347	DOWN	238.8915277	8.3484	2'760'781	35'319
49	77.42895396	UP	293.2133326	16.3613	0	1'416'113
50	76.02174354	DOWN	260.8730019	16.0884	1'390'377	0
51	75.89263658	DOWN	287.0559108	5.943483333	1'388'015	0
52	73.30690712	UP	292.1989361	14.9393	0	1'340'724
53	72.25317112	DOWN	269.0453145	8.7834	1'321'452	0
54	71.70347304	DOWN	238.8920945	13.14841667	3'200'738	44'639
55	71.59426158	DOWN	221.0831286	5.0884	1'309'401	0
56	69.56393395	UP	512.2898072	33.2083	0	1'272'268
57	69.18365047	DOWN	238.8922932	7.228466667	3'572'466	51'637
58	69.01546855	UP	265.148365	15.2658	0	1'262'237
59	57.07455379	DOWN	345.1719282	11.7484	1'043'848	0
60	56.39027684	UP	379.2103845	16.66496667	0	1'031'333
61	50.55290116	UP	445.1816665	14.96046667	0	924'572
62	49.8858403	DOWN	238.8929406	11.43846667	2'779'256	55'712
63	49.345539	DOWN	283.0618342	14.14840833	902'490	0
64	45.51860628	DOWN	345.207411	21.7584	832'499	0
65	44.74256847	DOWN	238.8928143	8.970933333	2'617'408	58'499
66	44.67913285	DOWN	237.0590608	10.6684	817'145	0
67	44.52327047	DOWN	345.062071	5.5284	814'295	0
68	44.51375826	UP	379.2088801	16.34496667	0	814'121
69	40.38702654	UP	289.1790365	13.97323333	0	738'646
70	40.32203764	DOWN	331.0454719	10.6684	737'458	0
71	40.28278504	DOWN	238.8919538	5.638466667	16'195'815	402'053
72	36.86149488	DOWN	260.8736651	15.6834	674'167	0
73	36.3050166	UP	316.9468272	20.0328	0	663'990
74	34.81207159	UP	289.1817254	14.27625	0	636'685
75	34.42563906	DOWN	265.3472795	37.6234	629'617	0
76	34.20221972	UP	310.2089283	14.9393	0	625'531
77	34.19950475	DOWN	238.8920297	12.1084	3'467'404	101'388
78	33.4637193	DOWN	299.0205731	10.6684	612'025	0
79	33.25462133	DOWN	238.8920107	9.290975	2'493'355	74'978
80	31.7556012	UP	312.2260566	16.66496667	0	580'784
81	29.98511105	DOWN	438.1431281	13.69841667	548'404	0
82	28.67964582	DOWN	309.1757243	21.6484	524'528	0
83	27.81261749	UP	355.1576525	23.77313333	0	508'670
84	27.78599846	DOWN	265.3481278	49.68846667	508'184	0
85	27.4922566	DOWN	444.9733963	15.48846667	502'811	0
86	26.12780309	DOWN	260.8736155	15.2234	477'857	0
87	24.08265054	DOWN	248.9603615	11.19843333	626'658	26'021
88	22.2354849	DOWN	238.8919572	6.6284	3'892'163	175'043
89	21.40368714	DOWN	238.8917313	6.3284	4'291'704	200'512
90	21.11629782	DOWN	452.925023	0.9084	386'200	0
91	20.92219893	DOWN	322.9111833	0.9084	382'650	0

92	20.43953457	DOWN	238.8923996	6.9434	5'786'961	283'126
93	20.33339356	DOWN	282.8548995	25.21346667	371'881	0
94	20.13679427	DOWN	265.3468191	6.0534	368'286	0
95	19.09196671	DOWN	265.347973	29.36346667	349'177	0
96	18.55684364	DOWN	265.3460362	25.58846667	339'390	0
97	18.38407508	DOWN	394.9784462	13.12343333	336'230	0
98	15.79210809	DOWN	375.0728169	7.713466667	288'825	0
99	14.8443893	DOWN	238.8918008	8.737266667	4'329'010	291'626
100	13.28924976	DOWN	238.8921201	44.46846667	243'050	0
101	13.0709959	DOWN	238.8921	42.8984	239'058	0
102	11.41841365	DOWN	238.8924449	46.31346667	208'834	0
103	10.9759242	DOWN	328.8614148	22.44846667	200'741	0
104	10.49061997	DOWN	302.0358733	8.3984	191'865	0
105	9.747095959	DOWN	260.8707913	46.41346667	178'267	0
106	8.480416736	DOWN	520.9089086	0.9084	155'100	0
107	8.155293633	DOWN	300.019802	10.7184	149'154	0
108	7.769820765	DOWN	238.8935053	49.47346667	142'104	0
109	3.704327073	DOWN	248.9602066	0.9084	1'082'406	292'200
110	1.813200522	UP	293.1760703	13.87341667	467'349	847'397
