# Supplementary material for: A bioactivity-leveling approach for component-specific evaluation in natural product extracts: Optimizing SARS-CoV-2 antiviral screening by controlling for pheophorbide A
Source: PLoS One. 2026 Aug 24;21(8):e0355466. doi: 10.1371/journal.pone.0355466 (PMC13502696; doi:10.1371/journal.pone.0355466)
Supplement: Supplementary file 8 [file pone.0355466.s011.pdf]

featureidx	fold	updown	mzmed	rtmed	dataset1_mean	dataset2_mean
1	11173.73515	UP	333.0651855	4.133833333	0	37'065'343
2	2758.322878	UP	303.0487061	4.233833333	0	9'149'866
3	2487.171674	UP	207.0252991	1.723833333	0	8'250'408
4	2047.068546	UP	334.0687866	4.133833333	0	6'790'504
5	1612.802709	UP	329.0314941	12.92383333	0	5'349'965
6	1379.507591	UP	302.0434875	8.183833333	0	4'576'082
7	844.8441185	UP	284.9380798	8.258833333	0	2'802'504
8	816.4044904	UP	393.0740051	12.89883333	0	2'708'165
9	721.5338013	UP	333.0651855	4.523833333	0	2'393'461
10	665.9823577	UP	311.184082	13.72383333	0	2'209'187
11	603.785339	UP	253.0513	8.433833333	0	2'002'868
12	560.8665653	UP	331.0452881	8.108833333	0	1'860'498
13	495.0045412	UP	441.1763	14.32383333	0	1'642'022
14	490.9474865	UP	285.1940918	8.283833333	0	1'628'564
15	489.7618425	UP	300.9412842	8.183833333	0	1'624'631
16	479.6897474	UP	401.0405884	4.133833333	0	1'591'220
17	462.2247869	UP	304.055603	4.258833333	0	1'533'285
18	427.2252286	UP	571.093689	8.308833333	0	1'417'185
19	388.7781686	UP	539.1105957	12.92383333	0	1'289'649
20	352.4785621	UP	335.056488	4.133833333	0	1'169'237
21	348.254388	UP	285.0340881	9.898833333	4'729	1'646'876
22	337.4771364	UP	435.1277771	15.74883333	0	1'119'474
23	328.4269922	UP	415.0296021	8.158833333	0	1'089'453
24	309.6755493	UP	285.0340881	9.573833333	0	1'027'251
25	308.0548979	UP	333.2207947	4.158833333	0	1'021'875
26	285.6994953	UP	330.0289917	12.92383333	0	947'718
27	270.7369481	UP	286.0426941	8.283833333	29'596	8'012'844
28	268.5157974	UP	329.237793	10.82383333	0	890'717
29	258.5925118	UP	208.0352936	1.698833333	0	857'799
30	256.6173042	UP	333.3764954	4.133833333	0	851'247
31	255.6948352	UP	333.4284058	4.133833333	0	848'187
32	252.0782142	UP	301.0398865	12.92383333	0	836'190
33	247.3360905	UP	399.0319824	8.508833333	0	820'460
34	234.0814774	UP	332.9440918	4.133833333	0	776'492
35	233.392791	UP	311.2174988	14.67383333	0	774'207
36	232.8346354	UP	417.0392761	4.358833333	0	772'355
37	231.7062806	UP	316.9598999	6.533833333	0	768'613
38	223.4436918	UP	399.0319824	8.258833333	0	741'204
39	221.7843598	UP	285.0340881	8.758833333	29'520	6'547'005
40	218.4338713	UP	301.3851929	8.183833333	0	724'586
41	209.3638307	UP	237.0797882	3.983833333	0	694'499
42	201.3346262	UP	415.0681763	1.723833333	0	667'864
43	196.5238765	UP	221.0789948	9.773833333	0	651'906
44	191.2391241	UP	369.0164795	8.183833333	0	634'376
45	189.3336106	UP	311.2174988	16.87383333	0	628'055

46	183.7675775	UP	366.128479	24.34883333	0	609'591
47	180.3474786	UP	385.0571899	6.533833333	0	598'246
48	179.8089183	UP	317.0610962	7.333833333	7'263	1'305'907
49	170.3289228	UP	394.0889893	12.89883333	0	565'013
50	170.1282808	UP	317.2297974	6.533833333	0	564'347
51	168.2208099	UP	285.9465027	8.283833333	0	558'020
52	163.715182	UP	353.032196	8.308833333	0	543'074
53	163.5352101	UP	330.1838989	17.61883333	0	542'477
54	157.9767718	UP	348.0831909	6.733833333	0	524'038
55	152.0405231	UP	271.225708	24.57383333	0	504'347
56	151.3771594	UP	253.0513	9.698833333	0	502'146
57	148.0865679	UP	572.090271	8.308833333	0	491'231
58	147.6027238	UP	409.240387	23.69383333	0	489'626
59	146.6879421	UP	359.0386963	10.24883333	0	486'591
60	144.4848691	UP	439.1488953	11.79883333	0	479'283
61	143.2924725	UP	287.1974792	20.11383333	0	475'328
62	140.9195698	UP	287.0529785	7.553833333	12'488	1'759'757
63	139.1044189	UP	347.0763855	6.75875	24'036	3'343'497
64	136.4042217	UP	299.0377808	12.87383333	0	452'478
65	129.7312439	UP	285.0340881	4.233833333	0	430'342
66	128.0884453	UP	447.0462036	4.458833333	0	424'893
67	126.2267662	UP	371.0393982	4.258833333	0	418'717
68	124.8059831	UP	347.0586853	10.24883333	0	414'005
69	121.32785	UP	333.0651855	5.048833333	0	402'467
70	110.5172099	UP	540.1229858	12.92383333	0	366'606
71	106.1507449	UP	285.0340881	9.748833333	9'103	966'279
72	100.9707623	UP	319.0717773	6.533833333	4'945	499'322
73	98.38264028	UP	285.0340881	8.342166667	456'315	44'893'475
74	97.68629951	UP	293.2174988	18.31883333	0	324'044
75	97.68449075	UP	333.1688843	13.72383333	0	324'038
76	97.39901775	DOWN	269.0456848	8.730166667	323'091	0
77	97.06323728	UP	371.0211792	1.048833333	0	321'977
78	96.85155552	UP	343.0462036	10.27383333	0	321'275
79	94.044808	UP	345.4367981	10.27383333	0	311'964
80	93.9136727	UP	299.0213928	12.64883333	0	311'529
81	93.0871238	UP	331.0452881	7.793833333	0	308'787
82	91.2466521	UP	301.9447021	8.183833333	0	302'682
83	88.57351787	UP	177.0222931	1.893833333	0	293'815
84	88.12080265	UP	549.4544678	34.28883333	0	292'313
85	87.19592802	UP	317.0274048	8.183833333	0	289'245
86	86.61610062	UP	345.2255859	10.27383333	4'963	429'861
87	85.01059624	UP	299.0541992	10.82383333	21'551	1'832'048
88	84.26959544	UP	431.0661926	7.333833333	0	279'538
89	84.20649546	UP	263.1266785	6.993833333	0	279'328
90	83.2930703	UP	316.0496826	12.89883333	0	276'299
91	81.18631394	UP	538.1212158	12.89883333	0	269'310

92	80.88560714	UP	253.0513	8.733833333	0	268'313
93	79.13382046	UP	555.1542969	14.32383333	0	262'502
94	74.78543865	UP	288.0651855	7.553833333	0	248'077
95	74.33335055	UP	267.0601807	11.04883333	9'133	678'903
96	73.25864615	UP	163.0401001	3.338833333	0	243'012
97	72.60780463	UP	331.0452881	10.34883333	0	240'854
98	72.32473327	UP	347.1998901	17.34383333	0	239'915
99	71.50616379	UP	525.4498901	35.80883333	0	237'199
100	70.77507582	UP	300.0216064	12.87383333	0	234'774
101	70.54927174	UP	284.0592041	8.483833333	7'569	534'023
102	70.06965837	UP	332.6330872	4.183833333	0	232'434
103	69.60155058	UP	547.4354858	32.75883333	0	230'881
104	69.22587056	UP	451.1217957	14.27383333	0	229'635
105	67.1679508	UP	315.0567932	14.99883333	0	222'809
106	67.01556254	UP	653.2544556	21.25883333	0	222'303
107	66.75826605	UP	396.0661926	4.158833333	0	221'450
108	65.88372928	UP	555.1542969	16.54883333	0	218'549
109	65.48625367	UP	327.2124023	9.848833333	0	217'230
110	64.78119797	UP	199.020401	1.338833333	3'317	214'891
111	63.21036764	UP	293.2174988	18.04383333	11'258	711'635
112	62.61800822	UP	445.0452881	8.108833333	0	207'716
113	60.91279718	UP	345.6129761	10.27383333	0	202'059
114	57.47815225	UP	299.0541992	11.09883333	24'028	1'381'073
115	57.47300532	UP	463.0925903	4.593833333	0	190'649
116	55.91026964	UP	285.0340881	12.89883333	45'618	2'550'540
117	54.13969587	UP	291.1932068	17.08133333	7'261	393'099
118	53.94774942	UP	541.1362915	8.058833333	0	178'955
119	51.92686407	UP	332.5984802	4.083833333	0	172'251
120	48.83749737	UP	347.0410767	4.133833333	0	162'003
121	47.41897522	UP	331.0452881	12.87383333	0	157'298
122	47.25889972	UP	509.1174011	12.64883333	0	156'767
123	47.04546572	UP	248.953598	11.47383333	0	156'059
124	46.5132373	UP	269.0456848	13.44883333	0	154'293
125	46.03527687	UP	288.0651855	5.798833333	12'528	576'726
126	45.36332771	UP	327.2980957	28.44383333	0	150'479
127	45.16071925	UP	442.1717834	16.57383333	17'906	808'644
128	44.93554403	UP	415.0488892	6.558833333	0	149'060
129	44.91790859	UP	453.0152893	4.133833333	0	149'001
130	44.79491273	UP	416.0338745	8.183833333	0	148'593
131	43.96152531	UP	364.028595	8.183833333	0	145'829
132	43.09196264	UP	330.2355957	10.82383333	0	142'944
133	42.80645191	UP	268.036499	8.483833333	21'295	911'544
134	41.78700613	UP	293.0228882	2.583833333	0	138'615
135	40.69083016	UP	343.0637817	12.87383333	0	134'979
136	40.20361701	UP	329.1690063	17.605	63'185	2'540'258
137	38.96549663	UP	385.0386047	7.578833333	0	129'256

138	37.79274011	UP	313.2431946	16.04883333	0	125'366
139	36.32719013	UP	604.0916138	8.183833333	0	120'504
140	35.92112291	UP	309.1984863	15.62383333	0	119'157
141	34.77391516	UP	299.9231873	10.39883333	0	115'352
142	34.53838693	UP	318.0740967	6.533833333	109'271	3'774'046
143	34.1926011	UP	299.3656006	10.27383333	3'483	119'088
144	34.08748972	UP	316.0159912	8.108833333	0	113'075
145	34.07416391	UP	299.398407	10.27383333	3'483	118'676
146	32.91869996	UP	313.0755005	12.14883333	10'374	341'507
147	32.67575043	UP	440.1619873	11.79883333	0	108'392
148	32.454177	UP	302.3399048	8.183833333	0	107'657
149	32.41183788	UP	315.0567932	12.92383333	50'944	1'651'196
150	31.46149788	UP	167.0323029	6.708833333	0	104'364
151	31.06457184	UP	328.9284058	12.89883333	6'720	208'742
152	30.60878685	UP	483.142395	13.57383333	0	101'535
153	30.41253609	UP	312.0538025	12.84883333	0	100'884
154	29.9951641	UP	409.1636963	13.72383333	0	99'500
155	29.92507455	UP	307.1860046	22.75883333	0	99'267
156	29.76997314	UP	328.0354919	12.89883333	0	98'753
157	29.72845199	UP	248.953598	19.76383333	0	98'615
158	29.41681223	UP	463.1537781	16.57383333	0	97'581
159	29.23076289	DOWN	470.1736755	16.61316667	96'964	0
160	28.78594399	UP	345.0671997	10.27383333	519'533	14'955'252
161	28.77153613	UP	329.0658875	11.34883333	0	95'441
162	28.68726588	UP	250.9906921	1.288833333	0	95'161
163	28.67883704	UP	277.02948	3.958833333	0	95'133
164	28.05421099	UP	361.0520935	8.058833333	0	93'061
165	27.8885434	UP	355.0466003	7.553833333	0	92'511
166	27.26004183	UP	317.0610962	6.564666667	767'308	20'916'837
167	26.73744191	UP	299.2016907	10.27383333	3'802	101'666
168	26.382899	UP	269.0456848	12.87383333	59'171	1'561'100
169	26.02726591	UP	299.0213928	8.242666667	35'979	936'435
170	25.4994844	UP	371.2219849	18.46883333	0	84'587
171	24.99393523	UP	344.4866028	10.24883333	0	82'910
172	24.89600519	UP	248.953598	17.16883333	3'494	86'996
173	24.63760897	UP	397.2171936	10.82383333	0	81'728
174	24.32243207	UP	429.2388	17.09383333	0	80'682
175	24.31791017	UP	302.3894043	8.183833333	0	80'667
176	23.88697245	UP	268.0675049	11.07383333	0	79'238
177	23.40968982	UP	346.0709839	10.28208333	127'959	2'995'481
178	23.25630753	UP	329.1690063	20.59883333	4'456	103'625
179	23.1932232	UP	346.1767883	11.62383333	8'984	208'362
180	23.02942742	UP	285.0340881	15.02383333	25'904	596'565
181	22.22926877	UP	295.2326965	19.68883333	43'965	977'303
182	22.15487114	DOWN	373.0495911	10.5	73'492	0
183	22.1482993	UP	288.0491028	10.17383333	0	73'470

184	22.08725356	UP	285.1940918	12.89883333	0	73'268
185	21.41439384	UP	395.0863953	12.89883333	0	71'036
186	21.39359306	UP	284.9380798	12.89883333	0	70'967
187	21.38183611	UP	169.0095978	10.17383333	0	70'928
188	21.22953829	UP	548.4334717	32.73383333	0	70'422
189	21.11323485	UP	435.1277771	19.68883333	0	70'037
190	20.74153411	UP	455.1956787	18.66883333	0	68'804
191	20.45077551	UP	312.2211914	16.87383333	0	67'839
192	20.25316818	UP	622.3673096	16.64883333	0	67'184
193	20.18276299	UP	269.0456848	14.99883333	11'523	232'569
194	20.15142528	UP	393.2619019	12.89883333	0	66'846
195	19.27417537	UP	272.2251892	24.57383333	0	63'936
196	19.04581908	UP	539.1105957	8.783833333	0	63'179
197	18.95583313	UP	556.1593018	16.54883333	0	62'880
198	18.42116288	UP	285.0180969	0.863833333	0	61'106
199	18.35261077	UP	654.2476807	21.28383333	0	60'879
200	18.1789901	UP	316.3360901	10.42383333	6'754	122'786
201	18.01211119	UP	414.0457764	10.29883333	0	59'750
202	17.70190839	UP	222.0802917	9.773833333	0	58'721
203	17.46043857	UP	351.2187805	15.04883333	0	57'920
204	17.45094256	UP	637.1312866	4.208833333	0	57'888
205	17.15294891	UP	420.995575	8.158833333	0	56'900
206	17.1289828	UP	429.2584839	17.09383333	0	56'820
207	16.5569616	UP	379.204895	14.67383333	0	54'923
208	16.43351354	UP	273.0386047	8.183833333	0	54'513
209	16.42040001	UP	271.0228882	10.39883333	0	54'470
210	16.36163738	UP	283.0541077	8.516166667	200'488	3'280'319
211	16.20787039	UP	373.0861816	12.87383333	0	53'765
212	16.12736237	UP	361.0160828	1.313833333	0	53'497
213	15.95871333	UP	346.0709839	12.89883333	0	52'938
214	15.73997321	UP	397.0283813	10.27383333	3'760	59'178
215	15.66329238	UP	349.0559998	6.533833333	4'918	77'034
216	15.61685718	UP	556.1593018	14.32383333	0	51'804
217	15.31443206	UP	526.4494019	35.80883333	0	50'801
218	15.18079464	UP	291.047699	7.128833333	0	50'358
219	15.09484826	UP	293.0228882	2.393833333	0	50'072
220	15.03759544	UP	287.0529785	6.108833333	18'806	282'793
221	14.90744747	UP	313.0419922	12.87383333	18'460	275'189
222	14.75317299	UP	452.128479	14.29883333	0	48'939
223	14.64057751	UP	311.0838928	13.72383333	0	48'566
224	14.6373694	DOWN	239.0678864	18.67766667	117'684	8'040
225	14.55330471	UP	318.4289856	6.508833333	0	48'276
226	14.54652185	UP	329.237793	15.67383333	0	48'254
227	14.37016749	UP	397.1605835	17.61883333	0	47'669
228	13.66659756	UP	297.2384033	20.28883333	0	45'335
229	13.46823	UP	391.0666809	10.69341667	23'348	314'450

230	13.3561299	UP	331.1141968	7.018833333	0	44'305
231	13.09589416	UP	286.4754944	8.283833333	0	43'442
232	12.91727885	UP	437.3241882	13.57383333	0	42'849
233	12.9000956	UP	555.1096802	12.89883333	0	42'792
234	12.80036829	DOWN	423.1560974	16.6435	2'154'878	168'345
235	12.56321355	UP	357.102478	9.548833333	0	41'675
236	12.4730829	UP	525.102478	6.943833333	0	41'376
237	12.3144397	UP	287.0529785	5.849833333	332'137	4'090'083
238	12.28556847	UP	432.0699768	7.333833333	0	40'754
239	12.15670306	UP	441.1763	16.57383333	219'804	2'672'088
240	12.12775393	DOWN	221.1212921	13.2135	40'230	0
241	11.75876633	UP	330.941803	8.108833333	0	39'006
242	11.58783826	UP	371.1306763	6.683833333	0	38'439
243	11.21568533	UP	339.2140808	24.57383333	0	37'205
244	11.20392838	UP	415.0681763	6.733833333	0	37'166
245	11.07208092	UP	272.0532837	9.948833333	3'551	39'321
246	10.88196861	UP	292.1963806	17.16883333	0	36'098
247	10.88151642	UP	255.0293884	10.42383333	0	36'096
248	10.88151642	UP	397.0473022	8.508833333	0	36'096
249	10.71043098	UP	309.1984863	11.94883333	7'540	80'757
250	10.70664525	DOWN	520.899292	0.770333333	35'516	0
251	10.18814533	UP	549.12146	5.688833333	0	33'796
252	10.11369507	UP	374.9000854	0.793833333	3'364	34'018
253	10.07098548	DOWN	425.165802	16.63816667	78'025	7'748
254	10.00601761	UP	313.0419922	10.39883333	19'674	196'863
255	9.976556268	UP	633.2631836	30.12383333	0	33'094
256	9.75510945	UP	318.5980835	6.533833333	0	32'360
257	9.664787592	UP	279.0516052	3.338833333	0	32'060
258	9.621894077	UP	413.197998	21.63383333	0	31'918
259	9.305265091	UP	413.0439758	10.29883333	55'559	516'990
260	9.046526661	UP	387.2167969	15.37383333	0	30'009
261	9.030408882	DOWN	272.0688782	9.461166667	342'216	37'896
262	8.958506348	UP	256.0441895	10.27383333	11'086	99'318
263	8.917200128	UP	509.1600952	16.57383333	0	29'580
264	8.913582602	UP	258.0187073	12.89883333	0	29'568
265	8.883918894	UP	293.0066833	1.048833333	0	29'470
266	8.6178499	UP	299.0541992	6.533833333	0	28'587
267	8.468988204	UP	294.1755066	13.74883333	9'280	78'593
268	8.341139047	UP	346.2120972	21.60883333	19'114	159'433
269	8.204547622	UP	399.2779846	19.76383333	0	27'216
270	8.079088592	UP	362.0610046	10.67383333	14'664	118'473
271	7.919668113	UP	315.9487	10.42383333	37'258	295'074
272	7.869606675	UP	315.5782776	10.42383333	81'543	641'715
273	7.543340263	DOWN	426.1821899	18.39216667	740'262	98'135
274	7.495060452	UP	285.5462036	12.89883333	0	24'863
275	7.474978691	UP	287.0369873	10.17383333	132'857	993'101

276	7.449208317	UP	350.0480042	7.578833333	0	24'710
277	7.437038156	UP	329.1861877	19.42366667	143'946	1'070'531
278	7.393769741	UP	685.3861084	20.11383333	0	24'527
279	7.173919841	UP	301.0316925	10.429	95'946	688'313
280	6.916479467	UP	301.0398865	8.326333333	464'078	3'209'783
281	6.705080055	UP	329.0314941	10.42383333	54'585	365'996
282	6.608766727	UP	270.9604797	9.923833333	0	21'923
283	6.516112858	DOWN	425.7911987	18.3905	21'615	0
284	6.42863205	UP	485.1644897	23.16883333	0	21'325
285	6.4108873	UP	315.4100037	10.4165	140'872	903'117
286	6.311677453	UP	349.1976013	13.89883333	0	20'937
287	6.253265635	UP	269.0456848	11.07383333	263'174	1'645'695
288	6.251053394	DOWN	425.1853027	18.39383333	2'299'541	367'865
289	6.211207377	UP	384.0354004	10.42383333	8'312	51'627
290	6.134979425	UP	637.2747803	25.71383333	0	20'351
291	6.014858652	DOWN	268.9523926	9.948833333	13'691'250	2'276'238
292	5.813401644	UP	282.0507813	11.09883333	14'083	81'872
293	5.690222764	UP	659.2005615	23.25883333	0	18'876
294	5.558600641	UP	315.2080994	10.4165	211'316	1'174'623
295	5.557902319	DOWN	346.0709839	8.018333333	56'248	10'120
296	5.345489571	UP	248.953598	25.96883333	11'594	61'974
297	5.267637933	UP	285.754303	10.17383333	75'640	398'444
298	5.226442023	UP	317.0610962	10.4165	200'637	1'048'620
299	5.214031912	UP	298.071991	11.09883333	40'618	211'785
300	5.173965713	UP	347.6413879	6.733833333	0	17'163
301	5.116360798	UP	301.1714783	11.59883333	14'975	76'620
302	5.064535569	UP	316.9260864	10.39883333	0	16'800
303	5.014012355	DOWN	298.0393066	13.18783333	49'917	9'956
304	4.988203339	UP	300.0545044	10.27383333	163'072	813'438
305	4.939378965	UP	315.0567932	11.0395	391'572	1'934'123
306	4.871708907	UP	221.0789948	5.00875	320'859	1'563'131
307	4.816323791	UP	284.0272827	10.27383333	154'955	746'312
308	4.622629255	DOWN	271.0541077	9.461166667	1'795'905	388'503
309	4.622587308	UP	438.1369019	13.57083333	143'072	661'361
310	4.620849913	UP	345.2079773	21.62475	191'276	883'858
311	4.497590842	DOWN	345.0671997	5.425	208'352	46'325
312	4.413450557	UP	286.5716858	10.17383333	12'085	53'336
313	4.37246549	UP	437.145874	13.57383333	603'922	2'640'630
314	4.371979422	UP	293.1688843	24.02883333	12'824	56'066
315	4.268486224	UP	299.0541992	10.25841667	1'266'132	5'404'467
316	4.252591819	UP	361.0520935	10.65666667	199'833	849'809
317	4.210980968	UP	314.9389954	10.38533333	271'107	1'141'628
318	4.14421868	UP	315.0567932	10.4165	8'735'703	36'202'662
319	4.092762184	UP	316.0496826	10.40458333	1'622'962	6'642'398
320	4.061632641	UP	300.0216064	10.42383333	639'814	2'598'690
321	3.940606551	UP	285.9465027	10.17308333	87'647	345'383

322	3.829691387	UP	345.1727905	11.605	264'050	1'011'230
323	3.714502856	UP	269.3721008	9.948833333	327'144	1'215'177
324	3.68944503	UP	271.0541077	9.948833333	268'925	992'186
325	3.609342811	UP	284.9380798	10.17308333	588'324	2'123'463
326	3.596897686	UP	359.1822815	17.24383333	17'141	61'653
327	3.465774346	UP	285.1940918	10.17308333	363'651	1'260'333
328	3.369066214	DOWN	328.8596802	0.751	57'720	17'132
329	3.315317788	UP	413.0246887	10.41708333	85'239	282'594
330	2.99522945	UP	269.1854858	9.948833333	484'179	1'450'227
331	2.953576762	UP	285.5462036	10.17383333	226'870	670'077
332	2.861010406	UP	286.651886	8.258833333	0	9'491
333	2.852029484	UP	270.0411987	9.948833333	2'370'674	6'761'234
334	2.742860088	DOWN	345.0671997	7.966083333	510'075	185'964
335	2.73273139	UP	330.9762878	13.07383333	29'624	80'954
336	2.700451698	UP	238.8920898	36.92383333	25'297	68'314
337	2.616885129	UP	299.0213928	10.53641667	158'077	413'669
338	2.608775861	UP	285.0340881	10.17308333	14'051'393	36'656'934
339	2.583454703	UP	286.0426941	10.16133333	2'417'511	6'245'529
340	2.5393221	DOWN	238.9067993	0.818833333	148'416	58'447
341	2.481296718	UP	269.0456848	9.948833333	15'662'153	38'862'450
342	2.444722361	UP	394.9733887	13.04883333	41'979	102'627
343	2.402294005	DOWN	375.0651855	7.623833333	63'881	26'592
344	2.400067416	UP	269.0456848	10.57383333	266'559	639'759
345	2.337088739	UP	331.0452881	10.57383333	187'056	437'166
346	2.300213592	DOWN	313.0755005	13.18783333	355'383	154'500
347	2.273541779	UP	329.0314941	10.65666667	446'487	1'015'107
348	2.256140467	DOWN	322.9078979	0.770333333	89'215	39'543
349	2.241250131	UP	283.0221863	10.17383333	90'974	203'897
350	2.185182301	UP	384.9270935	0.794333333	102'370	223'697
351	2.124895616	UP	269.0456848	11.77383333	150'280	319'329
352	2.063277447	UP	383.0333862	10.40458333	259'350	535'110
353	1.996479894	UP	260.872406	43.74383333	18'548	37'030
354	1.894157023	DOWN	265.3443909	37.87816667	146'153	77'160
355	1.858968723	UP	297.0749817	11.0395	453'836	843'666
356	1.839228402	UP	353.032196	10.17308333	268'961	494'681
357	1.79112661	UP	520.9208984	0.748833333	38'680	69'282
358	1.758127409	UP	238.8920898	3.944833333	765'484	1'345'818
359	1.700615032	UP	337.036377	9.948833333	260'804	443'528
360	1.684585633	UP	238.8920898	18.98166667	817'777	1'377'615
361	1.68362421	UP	377.0861816	7.938833333	62'004	104'391
362	1.646372514	UP	430.9678955	17.41883333	65'539	107'901
363	1.581653582	UP	269.0456848	10.28275	2'336'644	3'695'762
364	1.546198779	UP	248.953598	16.621	76'182	117'792
365	1.533563887	DOWN	260.872406	45.65733333	86'056	56'115
366	1.499171407	UP	494.9588013	17.4025	97'937	146'825
367	1.461449341	DOWN	260.872406	15.27166667	327'947	224'399

368	1.457408853	UP	293.1850891	23.95108333	25'831	37'646
369	1.450561465	DOWN	238.8920898	35.55716667	118'210	81'493
370	1.437489794	UP	283.0541077	13.95233333	174'536	250'893
371	1.4250302	UP	260.872406	21.01133333	217'868	310'468
372	1.413774575	DOWN	238.8920898	1.3275	4'315'794	3'052'675
373	1.411018195	UP	260.872406	18.54383333	281'623	397'376
374	1.399551566	DOWN	260.872406	15.54425	164'163	117'297
375	1.392990261	DOWN	238.8920898	2.643333333	430'295	308'900
376	1.391481495	DOWN	238.8920898	4.1535	746'007	536'124
377	1.375330656	DOWN	238.8920898	16.38333333	702'262	510'614
378	1.374369538	DOWN	238.8920898	2.203583333	375'304	273'074
379	1.352313297	DOWN	238.8920898	30.05683333	464'949	343'817
380	1.32942418	UP	309.1817932	21.52208333	142'678	189'680
381	1.321200053	DOWN	265.3443909	40.38083333	216'618	163'955
382	1.31202886	UP	383.0148926	10.14883333	63'348	83'115
383	1.297715213	DOWN	343.1514893	16.80641667	88'775	68'409
384	1.293621437	DOWN	238.8920898	11.06916667	974'177	753'062
385	1.274136764	DOWN	354.0297852	10.14883333	43'553	34'182
386	1.267071874	UP	265.3443909	44.22383333	114'838	145'508
387	1.255645962	UP	238.8920898	22.70883333	991'026	1'244'377
388	1.252058152	UP	260.872406	3.503833333	300'419	376'142
389	1.251106467	DOWN	238.8920898	3.603416667	568'527	454'419
390	1.238435146	DOWN	238.8920898	14.95383333	1'069'871	863'889
391	1.225349751	DOWN	238.8920898	4.3445	769'368	627'876
392	1.221726617	DOWN	238.8920898	3.011583333	635'203	519'923
393	1.21995003	UP	260.872406	19.90383333	168'517	205'582
394	1.199712243	UP	349.1268005	6.213583333	32'170	38'595
395	1.198304914	UP	265.1437988	19.38825	810'769	971'549
396	1.175603082	UP	316.9429932	0.768833333	176'448	207'432
397	1.173628924	DOWN	238.8920898	3.3235	581'539	495'505
398	1.17252366	UP	238.8920898	17.84383333	753'876	883'938
399	1.172326199	UP	238.8920898	24.67383333	793'391	930'113
400	1.169356179	UP	328.8596802	22.26483333	67'994	79'509
401	1.159349219	DOWN	238.8920898	6.57	920'024	793'569
402	1.149308765	DOWN	238.8920898	20.26933333	1'440'431	1'253'302
403	1.149117929	UP	260.872406	20.26383333	354'177	406'991
404	1.145757449	UP	444.9653015	15.363	123'406	141'393
405	1.142657884	UP	260.872406	20.55383333	214'373	244'955
406	1.132049432	DOWN	328.8596802	24.14883333	58'006	51'240
407	1.115977461	DOWN	260.872406	14.78083333	456'014	408'623
408	1.108357735	UP	238.8920898	23.69383333	816'546	905'025
409	1.102476477	UP	260.872406	20.73883333	234'321	258'333
410	1.10028014	DOWN	260.872406	14.3815	457'173	415'506
411	1.098608938	DOWN	238.8920898	11.28166667	656'016	597'134
412	1.095578427	DOWN	238.8920898	17.1025	778'575	710'652
413	1.093812967	UP	260.872406	18.91883333	310'543	339'677

414	1.07966175	DOWN	238.8920898	4.6515	778'299	720'873
415	1.076879755	UP	238.8920898	22.29883333	965'590	1'039'824
416	1.073934909	DOWN	260.872406	15.93383333	252'089	234'734
417	1.060885109	DOWN	238.8920898	21.779	872'767	822'678
418	1.059211918	UP	260.872406	19.19383333	309'601	327'933
419	1.056571692	DOWN	238.8920898	25.44883333	690'510	653'538
420	1.05491369	UP	260.872406	18.74383333	216'110	227'978
421	1.047522862	UP	260.872406	18.23133333	277'195	290'369
422	1.041930391	DOWN	383.0333862	9.948833333	263'076	252'489
423	1.039402816	UP	260.872406	19.46883333	248'977	258'788
424	1.037752936	DOWN	238.8920898	8.383833333	939'961	905'766
425	1.037655988	DOWN	238.8920898	23.49716667	838'495	808'066
426	1.037619937	DOWN	248.953598	11.0395	143'397	138'198
427	1.034516956	UP	238.8920898	23.92883333	814'010	842'107
428	1.032501018	DOWN	238.8920898	5.272166667	702'831	680'707
429	1.025519513	UP	238.8920898	5.060333333	864'109	886'161
430	1.024113455	DOWN	265.3443909	48.21383333	197'114	192'473
431	1.017621237	UP	238.8920898	23.04133333	751'103	764'338
432	1.01384689	UP	238.8920898	26.58883333	2'619'815	2'656'091
433	1.012782325	UP	238.8920898	26.01133333	1'806'387	1'829'477
434	1.011604841	DOWN	238.8920898	13.24883333	1'883'265	1'861'661
435	1.011587099	UP	429.0426025	10.40916667	475'172	480'678
436	1.010057291	DOWN	238.8920898	0.306166667	3'402'821	3'368'939
437	1.004698344	UP	238.8920898	12.82383333	597'869	600'678
438	1.004249932	UP	260.872406	16.37841667	165'885	166'590
439	1.00233206	DOWN	238.8920898	13.97383333	856'402	854'409
