# Supplementary material for: A bioactivity-leveling approach for component-specific evaluation in natural product extracts: Optimizing SARS-CoV-2 antiviral screening by controlling for pheophorbide A
Source: PLoS One. 2026 Aug 24;21(8):e0355466. doi: 10.1371/journal.pone.0355466 (PMC13502696; doi:10.1371/journal.pone.0355466)
Supplement: Supplementary file 10 [file pone.0355466.s013.pdf]

featureidx	fold	updown	mzmed	rtmed	dataset1_mean	dataset2_mean
1	801.436453	DOWN	425.2885826	27.30666667	32'244'381	0
2	564.6345135	DOWN	271.0581061	10.06666667	22'717'073	0
3	534.3313458	DOWN	269.0789246	10.36166667	21'497'879	0
4	434.6081562	DOWN	197.116676	4.146666667	17'485'692	0
5	261.0237821	DOWN	239.0683352	10.68166667	10'501'831	40233.23481
6	253.7266528	DOWN	300.0913646	11.21166667	10'208'244	0
7	208.8141717	DOWN	621.3063474	33.06666667	8'401'270	0
8	208.6066156	DOWN	287.0529952	10.28666667	8'392'919	0
9	198.798892	DOWN	579.1608735	12.38666667	7'998'323	0
10	185.7344689	DOWN	373.1266686	13.01166667	7'472'699	0
11	150.5117072	UP	379.2833643	38.859	0	6055572.857
12	132.0991557	DOWN	317.064294	10.53666667	5'314'776	0
13	131.5387148	DOWN	707.2085062	13.06166667	5'292'228	0
14	114.8861414	DOWN	279.0911928	12.38666667	23'696'402	206259.8779
15	93.55843964	DOWN	344.1197054	13.01166667	8'847'878	94570.5971
16	89.58583696	DOWN	299.0897169	11.21166667	56'754'065	633516.0381
17	72.69201728	UP	425.2873018	32.28533333	0	2924635
18	71.64766924	DOWN	270.0821971	10.36166667	2'882'618	0
19	68.61681095	UP	323.2211115	24.524	0	2760676.267
20	60.87429986	DOWN	647.1864058	13.83666667	2'449'170	0
21	51.12407362	DOWN	343.1151888	13.01166667	58'523'889	1144742.288
22	49.80511782	UP	351.2520716	29.99833333	0	2003821
23	39.38347436	DOWN	607.290242	32.00166667	1'584'525	0
24	39.15637629	DOWN	301.0727609	12.38666667	15'310'544	391010.2249
25	28.04855418	DOWN	367.0767907	11.76166667	5'464'970	194839.6329
26	19.23982508	UP	299.0940073	17.82166667	0	774080.4
27	16.08501685	UP	425.2147104	27.21083333	0	647152.26
28	15.40714954	UP	223.073217	13.94333333	2'901'081	44697388.8
29	12.61039455	DOWN	365.0972253	13.03666667	26'988'617	2140188.112
30	11.73149219	DOWN	657.2713546	30.28666667	471'996	0
31	10.80117177	UP	199.1347889	11.14	0	434566.08
32	5.947354667	DOWN	283.0945491	12.71166667	11'296'667	1899443.893
33	5.222770251	DOWN	314.1075598	13.57208333	9'752'628	1867328.55
34	3.028479157	DOWN	313.1053586	13.5825	51'079'836	16866497.45
35	2.477508583	DOWN	297.073779	14.4875	44'298'221	17880148.15
36	2.186398245	DOWN	284.0983925	14.88666667	11'337'774	5185594.174
37	1.603387478	DOWN	283.0949665	14.89966667	78'075'294	48693965.17
38	1.110496749	DOWN	253.083574	14.62475	52'187'631	46994852.58
39	1.098396946	UP	254.0875975	14.61166667	8'739'870	9599846.516
